# Evolution and function of developmentally dynamic pseudogenes in mammals

**DOI:** 10.1186/s13059-022-02802-y

**Published:** 2022-11-08

**Authors:** Sheng Hu Qian, Lu Chen, Yu-Li Xiong, Zhen-Xia Chen

**Affiliations:** 1 Hubei Hongshan Laboratory, College of Biomedicine and Health, Huazhong Agricultural University, Wuhan, 430070 PR China; 2 Hubei Key Laboratory of Agricultural Bioinformatics, College of Life Science and Technology, Huazhong Agricultural University, Wuhan, 430070 PR China; 3 Interdisciplinary Sciences Institute, Huazhong Agricultural University, Wuhan, 430070 PR China; 4 Shenzhen Institute of Nutrition and Health, Huazhong Agricultural University, Shenzhen, 518124 PR China; 5 Shenzhen Branch, Guangdong Laboratory for Lingnan Modern Agriculture, Genome Analysis Laboratory of the Ministry of Agriculture, Agricultural Genomics Institute at Shenzhen, Chinese Academy of Agricultural Sciences, Shenzhen, 518124 PR China

**Keywords:** Pseudogene, Developmentally dynamic expression, Mammals, Evolution, Iso-seq

## Abstract

**Background:**

Pseudogenes are excellent markers for genome evolution, which are emerging as crucial regulators of development and disease, especially cancer. However, systematic functional characterization and evolution of pseudogenes remain largely unexplored.

**Results:**

To systematically characterize pseudogenes, we date the origin of human and mouse pseudogenes across vertebrates and observe a burst of pseudogene gain in these two lineages. Based on a hybrid sequencing dataset combining full-length PacBio sequencing, sample-matched Illumina sequencing, and public time-course transcriptome data, we observe that abundant mammalian pseudogenes could be transcribed, which contribute to the establishment of organ identity. Our analyses reveal that developmentally dynamic pseudogenes are evolutionarily conserved and show an increasing weight during development. Besides, they are involved in complex transcriptional and post-transcriptional modulation, exhibiting the signatures of functional enrichment. Coding potential evaluation suggests that 19% of human pseudogenes could be translated, thus serving as a new way for protein innovation. Moreover, pseudogenes carry disease-associated SNPs and conduce to cancer transcriptome perturbation.

**Conclusions:**

Our discovery reveals an unexpectedly high abundance of mammalian pseudogenes that can be transcribed and translated, and these pseudogenes represent a novel regulatory layer. Our study also prioritizes developmentally dynamic pseudogenes with signatures of functional enrichment and provides a hybrid sequencing dataset for further unraveling their biological mechanisms in organ development and carcinogenesis in the future.

**Supplementary Information:**

The online version contains supplementary material available at 10.1186/s13059-022-02802-y.

## Background

Pseudogenes are defined as genomic regions that resemble functional genes, contain gene-disabling mutations, and lack regulatory elements required by transcription or translation [1]. Different pseudogenes are categorized based on their origination mechanisms: (1) unprocessed pseudogenes which are derived from segmental duplication and subsequent mutations; (2) processed pseudogenes which are formed through retrotransposition of processed mRNA; (3) unitary pseudogenes which are directly originated from inactivated functional genes through mutations; (4) polymorphic pseudogenes which segregate in the population both as a pseudogenised and an intact allele.

Moreover, pseudogenes are precious markers of genome remodeling and dynamics. This would be exemplified by processed pseudogenes which offer a perspective into the evolution of ancient transcriptome and activity of transposable elements [2–4]. The previous study has deduced the historical expression levels of the parent genes in human and mouse and found that 3% of them have been prominently changed during the evolution course [5]. Unprocessed pseudogenes disclose the gene duplication process which is the main source of the generation of new genes [6]. Nevertheless, pseudogenization is the eventual fate for the majority of duplicated genes, and certain copies can be retained in the genome to sustain ancestral function or obtain new function [7, 8]. Besides, unitary pseudogenes not only represent natural loss-of-function events that silence ancestral genes, but also elucidate gain-of-function mutations that confer novel function [9]. Polymorphic pseudogenes represent a relaxed selection, and they are highly likely to be fixed as unitary pseudogenes.

Since the “pseudogene” was first introduced to describe a truncated ribosomal gene in *Xenopus laevis* in 1977 [10], this term has been gradually regarded as genomic relics and non-functional fossils. Pioneering works have rehabilitated a processed *Adh* gene (*jingwei*) as a functional copy [11, 12], instead of a defective pseudogene as previously reported. With the availability of high-throughput sequencing recently, a growing body of evidence has uncovered the functions of some pseudogenes under physiological and pathological conditions [13–18]. For example, some studies showed that pseudogenes played important roles in cancer progression and could stratify the subtype of kidney cancer [19, 20]. Furthermore, pseudogenes were reported to exhibit tissue-specific expression, suggesting their distinct regulatory programs [21, 22]. Nevertheless, the studies of pseudogene transcription were precluded by the limited capacity of short-read sequencing. A recent work applied long-read PacBio sequencing to identify functional human pseudogenes and provided the evidence that pseudogenes regulated the cellular transcriptomes [23]. Although above findings significantly advanced our knowledge about pseudogene functions, the majority of these studies focused on the function of pseudogenes in the disease context. Also, most of them were limited to the interplay between pseudogenes and their parent coding genes like competing endogenous RNAs (ceRNA) behavior, where pseudogene transcripts could regulate parent mRNAs by competing for identical microRNAs [18, 24]. Moreover, only a subset of tissue expression data were covered in their analysis, which might unavoidably underrate pseudogene expression abundance due to the spatiotemporal gene expression pattern [25, 26]. Meanwhile, there were limited systematic functional characterizations of mammalian pseudogenes. In particular, the contribution of pseudogenes to organ development is largely unknown. Here, we thus systematically inferred the origin time of human and mouse pseudogenes and characterized their evolutionary pattern. Using PacBio full-length sequencing data, we identified full-length pseudogene transcripts. In combination with deep Illumina sequencing data and public developmental RNA-seq data [25], we dramatically expanded the analyzed dataset and profiled genome-wide pseudogene expression patterns. Additionally, we prioritized developmentally dynamic pseudogenes (DDPs) with signatures of functional enrichment, found that they might represent an additional regulatory layer, and determined their implications in disease. Coding potential evaluation showed that over 19% of all pseudogenes were translated and encoded potentially functional peptides. Taken together, our hybrid sequencing data and a multitude of expressed pseudogenes with functional features will provide resources and reference for determining biological relevance and biomedical application of these pseudogenes, especially the DDPs.

## Results

### Accelerated acquisition of pseudogenes in human and mouse lineage

**Fig. 1:**
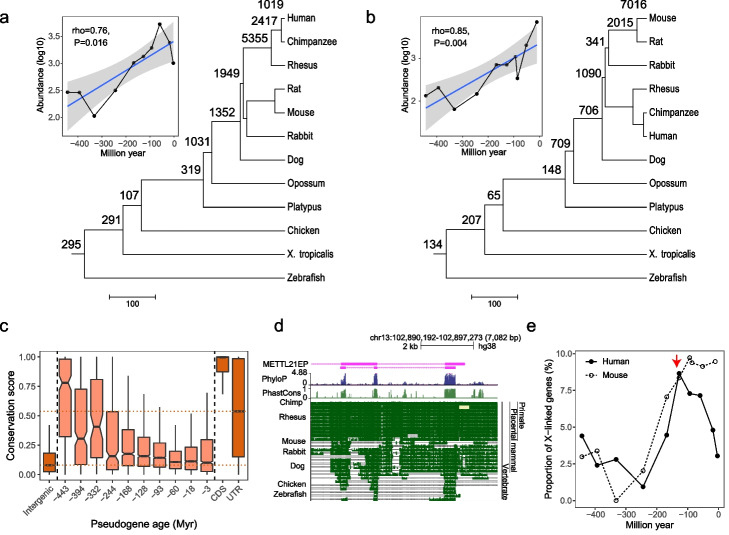
Phylogenetic distribution and genomic characteristics of pseudogenes. **a**, **b** show the assignments of pseudogenes to the branches of phylogenetic tree of human and mouse, respectively. Scatter plot represents the relationship between evolution branch and the corresponding number of pseudogenes in this branch. The evolutionary time (myr) is defined as the middle point of each branch. **c** Sequence conservations of randomly shuffled intergenic regions, pseudogenes with different ages, CDS, and untranslated terminal regions. **d** Genomic profile of the chromosome q33.1 locus. The enlarged picture depicts a highly conserved exon from the pseudogene *METTL21EP*. The Multiz alignment of 28 vertebrate species, the per-base phastCons, and the phyloP conservation scores are presented. **e** Proportions of X-linked pseudogenes originating in each phylogenetic branch

We dated the origin time of pseudogenes and assigned 14,136 human and 13,685 mouse pseudogenes annotated by GENCODE project [27] into different branches based on the presence and absence of orthologs in the vertebrate phylogenetic tree (Fig. 1a, b) (See “Methods”). In line with the previous report, we identified 2069 orthologous pseudogenes between human and mouse, and the sensitivity was similar (2069 vs 2237 from Gentree) [28]. Meanwhile, we observed the age distribution of pseudogenes with one peak at the dawn of primate lineage, which might mainly result from retrotransposition events (Fig. 1a) [29]. There was an accelerated acquisition of pseudogenes in rodent lineage (Fig. 1b), which was confirmed by one previous report on a recent successive burst of mouse processed pseudogenes based on the analysis of transposable elements [9]. The results recapitulated the above peak of pseudogenes when we only focused on processed pseudogenes (Additional file 1: Fig. S1). The larger proportion of inferred young pseudogenes might be due to fast sequence evolution. Alternatively, it might be due to the possibility that the phylogenetic distribution of species used skewed the distribution of estimated ages. To investigate these two possibilities, we examined the sequence conservation of human pseudogenes across vertebrates. We found that conservation was correlated with evolutionary age estimates and that the oldest pseudogenes had the highest levels of sequence conservation (median score ~ 0.78) (Fig. 1c), supporting that the recent gain of pseudogenes was owing to fast evolution. Notably, although young pseudogenes (evolutionary age < 18 myr) had lowest sequence conservation (median 0.1) among all pseudogenes with different ages, they still were more conserved than random intergenic regions (median 0.08, Wilcoxon test, *P* < 2 × 10^−16^), indicating the evolutionary constraint of these young pseudogenes. For example, *METTL21EP*, a duplicated pseudogene derived from methyltransferase *METTL21E*, originated prior to the divergence of tetrapods and teleost and harbored conserved exons in nearly all vertebrates including zebrafish (Fig. 1d).

We next tracked the chromosomal distribution of pseudogene within 450 myr prior to divergence between tetrapod and teleost, and observed one peak on the X chromosome (Fig. 1e, indicated by red arrow). This burst of pseudogenes occurred after the divergence of eutherian and marsupial (195 myr), which coincided with the first burst of protein-coding genes [30]. Besides, the contribution of this burst of X-linked pseudogenes to the genome (8.65% for human and 8.36% for mouse) was similar to that of protein-coding genes (8~14%). The consistency in time and size of burst between protein-coding genes and pseudogenes suggested that similar to protein-coding genes, some pseudogenes might have function, and this burst might be attributed to the emergence of X chromosome and subsequent recruitment of genes [30, 31]. Additionally, we found an accelerated accumulation of pseudogenes in rodent lineage instead of primate lineage, presumably due to the faster evolution of rodent genome [32]. Based on the distribution of the parent coding genes of pseudogenes, we found an excess recruitment of pseudogenes on the X chromosome (Fisher’s exact test, *P* < 1.6 × 10^−5^, Additional file 2: Table S1), which probably accounted for the burst of X-linked pseudogenes. Consistently, this gene recruitment pattern has also been reported in some previous studies of protein-coding genes [33, 34]. In all, above features including such nonrandom chromosomal distribution imply their functionality and inspire us for further analysis.

### Expressed pseudogenes contribute to organ identity

**Fig. 2:**
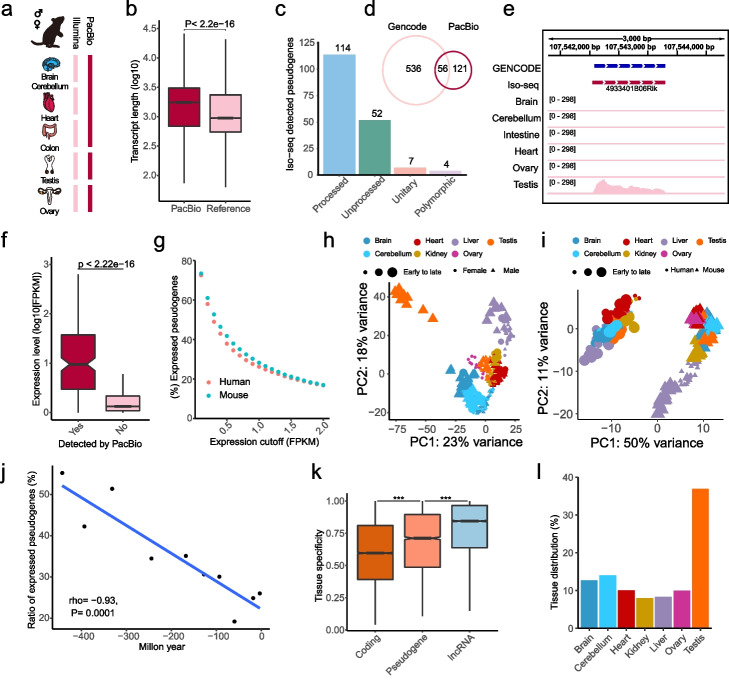
Tissue specificity of pseudogene expression. **a** Tissue samples from mouse. Somatic tissues were pooled into one sample for library preparation for each sex. **b** Length distribution of transcripts in ENSEMBL annotation and PacBio sequencing. **c** Biotype of pseudogenes detected by Iso-seq. **d** Venn plot shows the overlap between Iso-seq detected pseudogenes and transcribed pseudogenes annotated by GENCODE. **e** Expression pattern of Iso-seq detected pseudogene *4933401B06Rik*. **f** Expression level of Iso-seq detected pseudogenes and other pseudogenes. **g** Proportion of expressed pseudogenes under different thresholds. **h** Principal component analysis (PCA) of human pseudogenes. **i** PCA of 1:1 orthologous pseudogenes between human and mouse. **j** Proportion of human expressed pseudogenes with different evolutionary ages. **k** Tissue specificity of human pseudogene expression. **l** Distribution of the tissue with maximum expression level of human pseudogenes

Although the high-throughput RNA sequencing (RNA-seq) has revolutionized the manner of biological study, the short-read lengths hind their application in pseudogene transcriptome [1]. To accurately characterize and quantify the pseudogene transcription, we conducted PacBio Isoform Sequencing (Iso-seq) and sample-matched RNA-seq by using C57BL/6J male and female adult mouse tissues, including brain, cerebellum, heart, colon, and gonad (Fig. 2a). As expected, the transcripts detected by Iso-seq were longer than those annotated by ENSEMBL (Fig. 2b). We identified 177 pseudogenes, each of which was supported by at least one full-length read (Additional file 3: Table S2). Among them, 114 (64%) were processed pseudogenes (Fig. 2c). In line with previous report [23], most transcribed pseudogenes identified by Iso-seq were absent from GENCODE annotation and only 56 (31.6%) pseudogenes were shared between them (Fig. 2d). Illustrative examples were the transcribed pseudogenes *4933401B06Rik*, *Gm13857*, and *4632415L05Rik*, which do not overlap with any other gene structures. The first two were exclusively expressed in testis, while the third one (*4632415L05Rik*) exhibited a higher expression level in brain and ovary than in other tissues (Fig. 2e, Additional file 1: Fig. S2). A small number of pseudogenes was detected by Iso-seq, which might be due to the high tissue specificity of pseudogenes and the low throughput of Iso-seq [1]. Therefore, we combined our RNA-seq data with developmental transcriptome data [25] and integrated Iso-seq transcripts with ENSEMBL-annotated transcripts to systematically characterize pseudogenes. Interestingly, the pseudogenes detected by Iso-seq (median FPKM value, 8.5) showed significantly higher expression level than other pseudogenes (median, 0.33) (*P* = 2.2 × 10^−16^) (Fig. 2f). To further evaluate the accuracy of quantification, we compared the expression level between pseudogenes and their parent coding cognates and found that the correlation between them was negligible (Additional file 1: Fig. S3), which was consistent with the previous study [20]. Given that most pseudogenes have no expression, the expression correlation between pseudogenes and coding genes might be underrated when using all pseudogenes. Therefore, we performed the same analysis with only expressed ones and still observed only weak expression correlation (Additional file 1: Fig. S4). We further examined the correlation between these two types of genes in different tissues separately and obtained the similar results (Additional file 1: Fig. S5), demonstrating that the pseudogenes have acquired independent transcription programs. Given that the abundance of rRNA and mRNA led to the relatively small number of sequencing reads in pseudogenes and other non-coding RNAs, we applied a series of expression cutoffs to estimate the proportion of expressed pseudogenes (Fig. 2g). Our data showed that even we set FPKM ≥ 2 as cutoff, the proportion of detected pseudogenes was far higher than that pseudogenes expressed in human (16.8 vs 10%) and mouse (17 vs 5%) in ENSEMBL [35]. We next determined whether the expression of these pseudogenes was non-autonomous or function-driven. We performed a principle component analysis (PCA) and found that the tissue samples at different development stages clustered by the germ layer from which the tissues originate in human (Fig. 2h). In mouse, the tissues at early stage were clustered, suggesting strong commonalities, while these tissues gradually showed divergence with development (Additional file 1: Figs. S6-S7). The mature testis was separated from other tissues in both human and mouse. These observations suggested that the expressed pseudogenes contributed to organ development and identity. Although there was no noteworthy difference in pseudogene expression between male and female, the pseudogenes contributed considerable proportion to sex differential transcriptome (Additional file 1: Fig. S8). To our surprise, PCA analysis of orthologous pseudogenes separated the samples by species (50% explained variance) (Fig. 2i), and differences in organs and developmental stages among samples were less striking. Such pattern of pseudogenes was different from that of protein-coding genes (Additional file 1: Fig. S9) [25], but similar to that of lncRNAs [36], indicating rapid evolution of pseudogene expression.

Besides, the expression proportion of pseudogenes exhibited an age-dependent manner (Fig. 2j), suggesting a gradual acquisition of expression regulation after birth and the old pseudogene preservation in the genome induced by selective constraints at the transcriptional level. We further investigated pseudogene expression by origination mechanism and found that a higher proportion of pseudogenes were expressed in unitary type than in other types in both human and mouse (Additional file 1: Fig. S10), implying that some of them retained residual transcriptional activity. Although large fraction of expressed human unitary pseudogenes were detected based on the expression dataset covering successive development stages across multiple tissues, few vomeronasal and olfactory receptor-related unitary pseudogenes were expressed (3 out of 39, Fisher’s exact test, *P* = 2 × 10^−11^), demonstrating permanent loss of some olfactory function in human at the transcriptional level, which corroborated the previous observations at the DNA level [37]. In addition, the pseudogene-parent coding genes showed pronouncedly higher expression levels than non-pseudogene-generating coding genes in all tissues and developmental stages, which was more remarkable when only processed pseudogenes were considered (Additional file 1: Fig. S11), implying that the pseudogenes were more likely to be generated by highly expressed genes. These results were in line with previous studies [3, 9]. In addition, there was no difference in the generation of pseudogene between X chromosome (1.9 per protein-coding gene) and autosomes (2.1 per protein-coding gene) (*P* = 0.72), which agreed with our previous report of balanced expression level between X chromosome and autosomes [38].

**Fig. 3:**
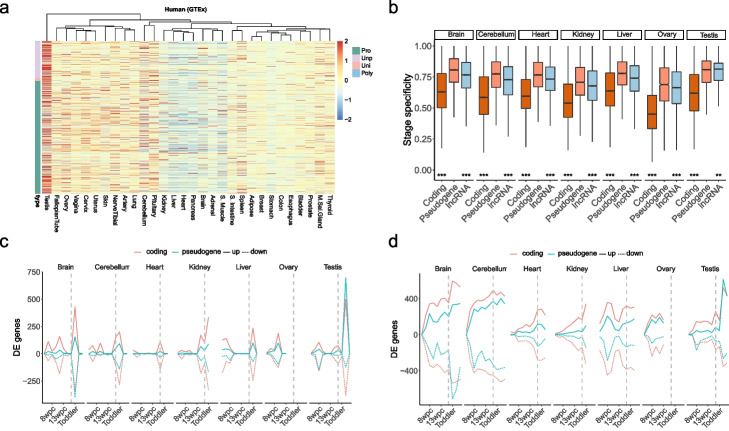
Development stage specificity of pseudogene expression. **a** Heatmap for human pseudogenes expression level based on GTEx data. **b** Developmental stage specificity of pseudogene expression. Protein-coding genes and lncRNAs are compared with pseudogenes separately. *, *P* < 0.05; **, *P* < 0.01; ***, *P* < 0.001. **c** Number of differentially expressed (DE) protein-coding genes and pseudogenes between adjacent developmental stages. Positive and negative values indicate upregulated and downregulated genes, respectively. To make the trend of pseudogenes clear, the number of protein-coding genes is divided by 10. **d** Number of DEGs in pairwise comparisons, referenced to tissues the 4th week post conception. The number of protein-coding genes is also divided by 10

To gain a preliminary expression profile of pseudogenes, we applied two indexes: (1) tissue specificity, which was to determine whether a pseudogene was broadly expressed across tissues or expressed tissue specifically; (2) developmental stage specificity, which was to determine whether a pseudogene in a certain tissue was expressed only at a specific developmental stage or successively expressed throughout development. In coincidence with previous study [21], the pseudogenes showed higher tissue specificity than protein-coding genes in human and mouse (Wilcoxon test, *P* < 2.2 × 10^−16^) (Fig. 2k, Additional file 1: Fig. S12). It should be noted that the tissue specificity of pseudogenes was significantly lower than that of lncRNAs, suggesting the strong tissue specificity of lncRNAs. We further explored the distribution of tissues in which highest pseudogene expression was observed, and we found that the pseudogenes pronouncedly preferred testis in both human and mouse (Fig. 2l, Additional file 1: Fig. S13), which might be due to the leaky expression caused by extensive chromatin remodeling [39]. In mouse, over 13% of pseudogenes were distributed in the liver and brain. The accuracy of the observations would increase with the larger number of samples. To validate our results, we integrated multiple datasets into our analysis, including Genotype Tissue Expression (GTEx), ENCODE, and an RNA-seq data containing those of about 30 healthy human tissues [40–42]. The results illustrated that human testis was the most distinctive tissue (Fig. 3a, Additional file 1: Fig. S14) with the significantly higher expression level of pseudogenes (35% ) than the other tissues, which might be due to the biological relevance of pseudogene to the testis, alternatively due to accessible chromatin environment of testis [26]. Interestingly, the mouse pseudogenes were preferentially expressed in all brain-related tissues, followed by placenta, liver, and testis, suggesting potential roles of pseudogenes in mouse brain development and evolution (Additional file 1: Figs. S15-S16). As for developmental stage specificity, we observed that pseudogenes showed the higher stage specificity than protein-coding genes and lncRNAs across nearly all tissues in both human and mouse (Fig. 3b, Additional file 1: Fig. S17). Overall, the strong spatiotemporal expression specificity of pseudogenes demonstrated strict transcription regulation programs rather than non-autonomous expression, which provided a new strategy for the development of biomarkers.

### DDPs mirror developmental trajectory

To get a comprehensive understanding of pseudogene expression during development, we implemented pairwise differential expression analysis to determine the stage with the greatest differential expression. Remarkably, the periods when pseudogenes showed the greatest differential expression coincided with those periods with the greater transcriptional changes (Fig. 3c), and the periods were related to the establishment of organ identity and organ-specific functions during development [25]. Although compared with differentially expressed (DE) coding genes, fewer DE pseudogenes were detected between neighboring stages, more DE pseudogenes were detected when comparing the older stages with 4 weeks post-conception (Fig. 3d), as observed for protein-coding genes, indicating that gradual and cumulative changes in pseudogene expression were identifiable only after sufficient time.

**Fig. 4:**
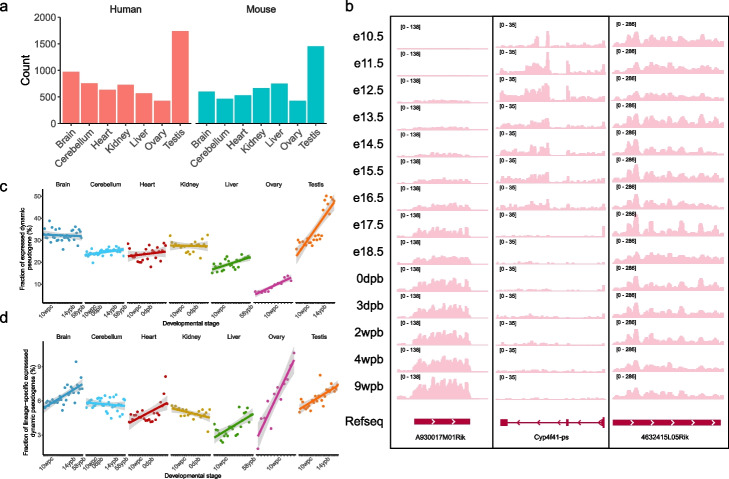
Expression patterns of dynamic pseudogenes. **a** Number of dynamic pseudogenes in human and mouse. **b** Representative IGV views of expression level of two DDPs in brain, *A930017M01Rik* and *Cyp4f41-ps*, and a non-dynamic one as control, *4632415L05Rik*. “e,” “dpb,” and “wpb” means embryonic day, day post-birth, and week post-birth, respectively. **c** Proportion of expressed dynamic pseudogenes during tissue development. **d** Proportion of lineage-specific expressed dynamic pseudogenes

To prioritize functional candidates, we next investigated DDPs with significant differential expression throughout development based on a regression approach [43]. Although dynamic expression is not essential for a functional transcript, we ratiocinated that it allows functionally relevant pseudogenes to be enriched, as some studies suggested [44–46]. Further, we identified 2741 and 2283 DDPs in human and mouse, respectively (Fig. 4a), and the proportion of DDPs (18%) was similar between the two. The comparable proportion of DDPs to that of developmentally dynamic lncRNAs (16–38%) [26] demonstrated equivalent functional importance of pseudogenes to that of lncRNAs, which has been largely ignored. The majority of the DDPs were processed pseudogenes, accounting for 56.8% (1558) human DDPs and 77.0% (1758) mouse DDPs. Representative IGV views of DDPs were displayed in Fig. 4b, a processed pseudogene *A930017M01Rik* exhibited gradually increased expression abundance during brain development while another unprocessed pseudogene *Cyp4f41-ps* were only expressed at early stages. We showed a non-dynamic pseudogene *4632415L05Rik* as control, which was broadly expressed among all stages investigated. We also analyzed the expression correlation between DDPs and their parent coding genes (Additional file 1: Fig. S18) and found that the median positive and negative correlation coefficient (*R*) was 0.33 and −0.08, respectively. To avoid any potential mis-mapping, we excluded 254 DDPs with a high correlation coefficient (*R* ≥ 0.6) from further analyses.

The transcriptomes exhibited strong similarity among different tissues at the earliest stages, and then showed increasing molecular and morphological differences with development [26, 47]. In agreement with such differences in development programs, the number of expressed DDPs was gradually increased in most tissues (Fig. 4c). Moreover, the proportion of recently evolved pseudogenes that might confer lineage-specific innovation was increased with time (Fig. 4d), which was consistent with one previous study of protein-coding genes [48]. Taken together, the expression of pseudogenes recapitulated gene expression programs during tissue development, suggesting that they might play roles in timing of gene expression.

### DDPs represent an additional regulatory layer

**Fig. 5:**
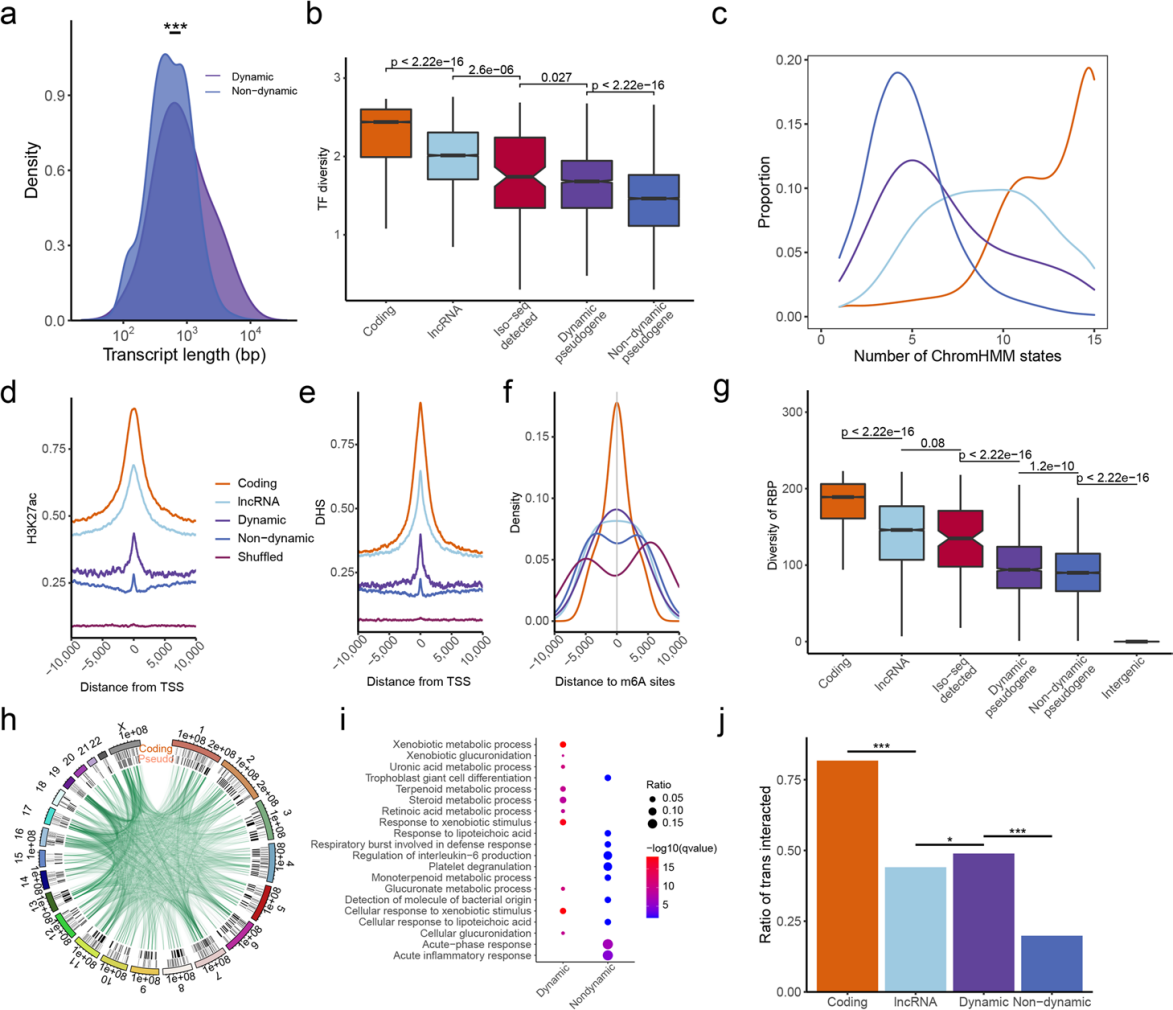
Regulatory layer of dynamic pseudogenes. **a** Distribution of transcript length for dynamic and non-dynamic human pseudogenes. **b** Diversity of TF binding sites overlapping the promoters of protein-coding genes, lncRNAs, Iso-seq detected dynamic pseudogenes, dynamic pseudogenes, non-dynamic pseudogenes, and randomly shuffled intergenic regions in mouse. **c** Number of chromHMM states overlapping protein-coding genes, dynamic pseudogenes, and non-dynamic pseudogenes. **d**, **e** Roadmap ChIP-seq signal of H3K27ac and DNase I hypersensitivity (DHS) at 10-kb intervals surrounding TSSs, respectively. **f** Density distribution of the distance from m6A modification sites to TSSs. **g** Number of RNA-binding proteins (RBPs) overlapping the promoter regions. **h** Circos plot showing genome-wide pseudogene–protein-coding gene contacts based on their pairwise-interacting RNAs. The first track (shown by coding) indicates protein-coding genes, and second track (shown by pseudo) represents pseudogenes. Green line, interaction between protein-coding genes and non-dynamic pseudogenes; grey line, interaction between protein-coding genes and dynamic pseudogenes. **i** GO enrichment (biological processes) of protein-coding genes significantly correlated with dynamic and non-dynamic pseudogenes. **j** Proportions of four types of genes interacting with mRNAs

Considering the potential role of DDPs in organ development, we then characterized these DDPs and investigated their functional clues. We found that the transcripts of DDPs were longer than those of non-dynamic pseudogenes (Fig. 5a, Additional file 1: Fig. S19), suggesting natural selection against premature polyadenylation signals to harbor more function RNA domains. By contrast, there was no difference in parent coding genes between DDPs and non-dynamic pseudogenes (Additional file 1: Fig. S20), suggesting longer length of DDPs was not conferred by their parent coding genes. Given that transcription factors (TFs) can cooperate with epigenetic modifications to remodel local chromatin state [49] and direct organ development, we further determined whether there was interplay between TFs and DDPs. As expected, significantly more abundant and diverse TFs bound to the promoters of DDPs than to those of non-dynamic ones and to randomly shuffled intergenic regions (Fig. 5b, Additional file 1: Figs. S21-S22), indicating a more complex transcriptional regulation. An expository locus was a DDP, *1700031M16Rik*, which showed a remarkable enrichment of TF binding sites at it proximal promoter region (Additional file 1: Fig. S23). Surprisingly, we observed that DDPs detected by Iso-seq showed more diverse TFs binding than other DDPs. Moreover, DDPs were covered by more chromHMM epigenetic signals (median 6) than non-dynamic ones (median 4) (Wilcoxon test, *P* < 2 × 10^−16^) (Fig. 5c), but they were overlapped less with quiescent state (15_Quies) (Additional file 1: Fig. S24), suggesting that diverse epigenetic states contributed to the dynamic expression of DDPs as well. To further validate the active transcription of DDPs, we intersected transcription start sites (TSSs) with ChIP-seq data including histone 3 lysine 27 acetylation (H3K27ac) and DNase I hypersensitivity sites (DHS). More H3K27ac and DHS signals were enriched at TSSs of DDPs than at TSSs of non-dynamic pseudogenes and randomly shuffled regions (Fig. 5d, e), suggesting that the DDPs possessed actively regulatory enhancers or promoters. Besides, the TSSs of DDPs were closer to m6A modification signals (Fig. 5f), and the transcripts of DDPs carried more RNA-binding proteins (Fig. 5g), alluding to the involvement of DDPs, especially Iso-seq detected DDPs, in post-transcription regulation or acting as scaffolds for RNA-binding proteins. We also observed the DDPs were more conserved than non-dynamic ones and exhibited a remarkable increase in the proportion of evolutionarily old pseudogenes (Spearman correlation rho = 0.94, *P* < 2 × 10^−16^) (Additional file 1: Figs. S25-S26), indicating that it took time, albeit short, for pseudogenes to acquire dynamic expression and to interact with more genes, thus integrating into pre-existing networks after birth [50, 51]. Overall, the above patterns across multiple regulatory layers provided functional evidences for DDPs.

We further mined two metrics to elucidate the function of these dynamic pseudogenes. First, we constructed a co-expression network between pseudogenes and protein-coding genes, via which functional associations or regulatory relationships could be inferred. The network connectivity relied on several factors such as increased functional interaction and expression abundance. Due to the accessible chromatin context and disproportionate RNA expression in adult testis [52], we excluded testis samples in this analysis. At strict *P* < 0.01 and absolute Pearson correlation coefficient *R* > 0.90, a total of 10,623 co-expression pairs were identified between 1268 coding genes and 463 pseudogenes (Fig. 5h, Additional file 4: Table S3). As expected, transcripts of DDPs were connected with more mRNAs than non-dynamic ones (Additional file 1: Fig. S27). The DDPs were significantly enriched in the metabolic process, meiotic process, and DNA modification, while non-dynamic pseudogenes were enriched in the inflammatory response and regulation of immune cells (Fig. 5i, Additional file 5: Table S4, Additional file 6: Table S5), indicating different functional properties between them.

Since the potential spurious co-expression might be generated, we then took advantage of RNA in situ conformation sequencing (RIC-seq) data to directly detect RNA–RNA interactions in vivo [53]. We observed that 1093 RNAs with *trans* interactions were from dynamic loci, accounting for 48.9% of all DDPs, which was significantly higher than that of non-dynamic ones (2377, 19.9%) (Fig. 5j) (Fisher’s exact test, *P* < 2.2 × 10^−16^). The DDPs possessed markedly more interacted mRNAs (an average of 7), compared with non-dynamic pseudogenes (average, 3), suggesting again that DDPs were involved in extensive and intricate transcription regulation. Three of the top 5 *trans* interacted RNAs from pseudogenes (connectivity > 90) were dynamic, including *AC004980.7*, *SUZ12P*, and *GUSBP1*. *GUSBP1* had large structural variation in HepG2, and its copy number variation was observed in 14% of colorectal cancer patient cohort [54, 55]. Besides, the hazard ratios of copy number variation within *GUSBP1* fluctuated over time and exhibited a significant association only early after diagnosis (first ~3 years), indicating that *GUSBP1* was a potential early-relapse biomarker.

### DDPs are more likely to be translated

**Fig. 6:**
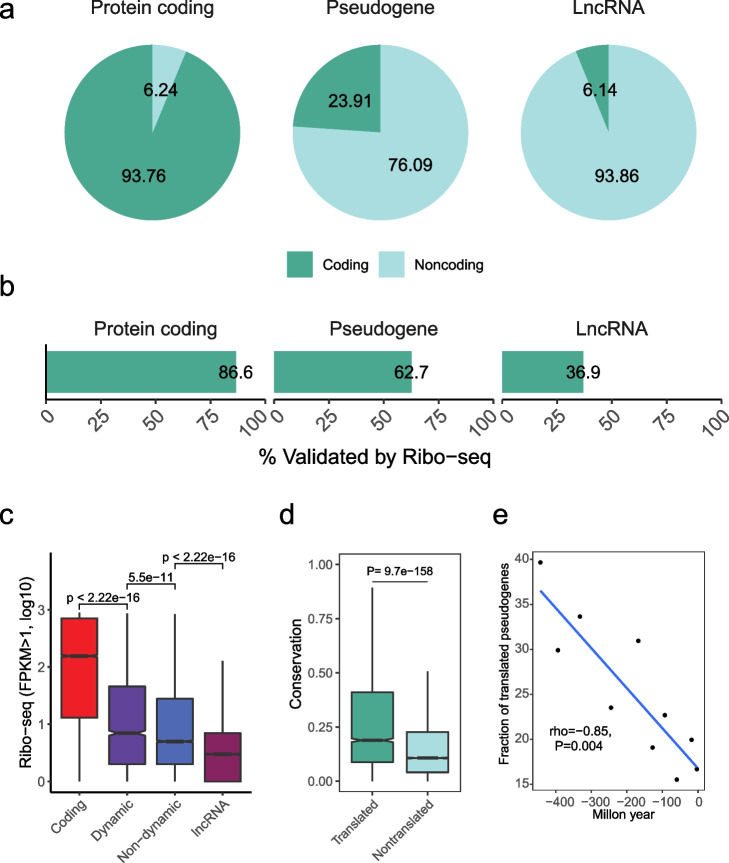
Coding potential of pseudogenes. **a** Proportion of translated RNA candidates of protein-coding genes, pseudogenes, and lncRNAs predicted by CPC2 and CPAT. **b** Proportion of translated RNA candidates validated by ribo-seq data. **c** Translation level of protein-coding genes, (non-)dynamic pseudogenes, and lncRNAs in Ribo-seq data. **d** Sequence conservation of translated and non-translated pseudogenes. **e** Proportion of translated pseudogenes with different evolutionary ages

In sporadic cases, several translated pseudogenes were identified from human by proteomics data [56–59] and their evolutionary constraints were observed [60]. We speculated that some pseudogenes might have the ability to generate “pseudoproteins,” but few pseudoproteins were actually detected due to the limited coverage and resolution of proteomic mass spectrometry (MS). The ribosome-profiling technique with high sensitivity made up for the shortage of MS method. One previous study evaluated the translation of pseudogenes with ribo-seq data and identified 426 and 81 expressed pseudogenes with their translated peptides longer than 10 and 100 aa, respectively [61]. This work might underestimate the number of expressed pseudogenes since only a limited number of cell lines were investigated. To gain a deep insight into pseudogene translation, we combined in silico prediction and public ribo-seq data to assess their coding capability. First, we subjected 15,244 human pseudogenes to CPC2 [62] and CPAT [63], and a pseudogene with at least one transcript passing the above two filters was treated as a translated candidate. To ascertain our results, we set protein-coding genes and lncRNAs as a positive or negative control, respectively. As expected, about 94% of protein-coding genes were identified as translated candidates, whereas only 6% of lncRNAs were identified (Fig. 6a), indicating that the non-coding dataset was well-annotated and that some lncRNAs were able to encode functional peptides [64]. Meanwhile, we found that 3645 (24%) pseudogenes were identified as potential translated candidates. The higher translation potential of pseudogenes than lncRNAs (Fisher’s exact test, *P* < 2.2 × 10^−16^) could be retained from their parent protein-coding genes. If a pseudogene harbored an open reading frame generating proteins or peptides, it would be occupied by the ribosome and captured by ribosome-profiling experiments when the tested samples were large enough. Accordingly, we collected public ribo-seq data to validate these translated candidates. The mapping specificity of Ribo-seq short reads to pseudogenes could be exemplified by two loci, *AL589987.1* (DDP) and *NDUFB4P8* (non-dynamic), to which 18 and 15 unique mapped reads (MAPQ > 30) were aligned, respectively (Additional file 1: Figs. S28-S29). Among those potentially translated pseudogenes, 2941 of them exhibited the FPKM ≥ 1.

To further screen the actively translated pseudogenes instead of randomly co-purified with the ribosome, we collected 24,724,526 transcripts with actively translated open reading frames (ORF) detected by RibORF [61], which used Ribo-seq data and combines alignment of ribosomal A-sites, characteristic 3-nt periodicity, and uniformity across codons. The pseudogenes with FPKM ≥ 1 in ribo-seq data and harbored at least one ORF were considered to be translated. Among the 2941 pseudogenes with FPKM ≥ 1, 2286 of them contained at least one active ORF (Fig. 6b) (Additional file 7: Table S6), accounting for 15% of all pseudogenes, notably surpassing previous estimates (4, 155, 140, and 272 pseudogenes reported to be translated, respectively in 4 different studies) [35, 57, 61, 65]. Besides, GENCODE annotated four translated pseudogenes using MS data (*AC113404.3*, *PMS2P2*, *AC092128.1*, and *CYP2G1P*) and a recent study generated a quantitative proteome across 29 human tissues and detected peptide evidence of four pseudogenes (*WASH9P*, *GPX1*, *GBA3*, and *PNLIPRP2*) [41]. Among the 8 translated pseudogenes with peptide evidence, 4 (50%) of them were determined as translated in our analysis (*AC113404.3*, *GPX1*, *GBA3*, and *PNLIPRP2*).

However, one previous study has revealed that translation per se cannot ensure functionality based on the comparison between synonymous and nonsynonymous changes [60]. To address this issue, we investigated the relationship between translation and expression dynamics since the DDPs were enriched with functional features. The DDPs were covered by significantly more ribosome fragments than non-dynamic ones (Fig. 6c), and non-dynamic pseudogenes still contained more reads than lncRNAs, demonstrating that a number of translated pseudogenes might have function. Next, we compared the sequence conservation of pseudogenes and found that the translated pseudogenes validated by ribo-seq were more conserved than others (Fig. 6d). Moreover, we observed a remarkable increase in the proportion of translated pseudogenes in older groups (Fig. 6e), suggesting a gradual acquisition of coding capability for pseudogenes and subsequent functional constraints on the preservation of old translated ones in the genome.

### DDPs conduce to cancer transcriptome alterations

To survey the association between pseudogenes and disease-associated regions, we overlapped pseudogenes with 141,418 unique disease-associated SNPs from the GWAS Catalog [66]. Pseudogene transcripts contained 3538 SNPs (0.07/kb). Of them, 2762 SNPs (0.18/kb) were located in their promoter regions, and the enrichment of SNPs suggested that these SNPs could play regulatory roles in their expression. In addition, relative to 65% of protein-coding genes overlapped with SNPs, 15.1% of DDPs harbored at least one SNP, which were significantly higher than non-dynamic ones (4.6%) (Fisher’s exact test, *P* < 2.2 × 10^−16^).

**Fig. 7:**
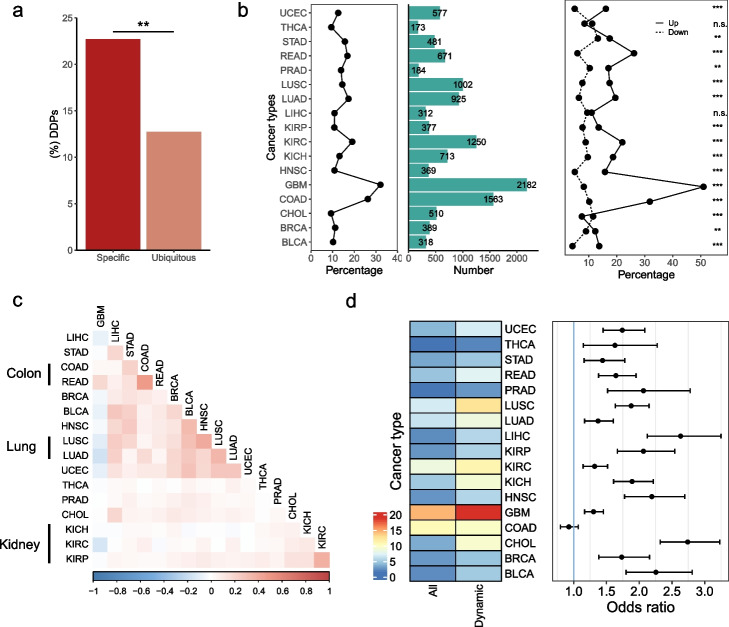
Differentially expressed pseudogenes in various cancer types. **a** Proportion of DDPs in cancer type-specific and ubiquitously expressed pseudogenes. **b** Proportion of differentially expressed (DE) pseudogenes in 17 cancer types. The left and middle panels represent the proportion and number of DE pseudogenes, respectively. The right panel indicates the proportion of up- and downregulated pseudogenes, respectively. **c** Correlation coefficient in pseudogene expression changes between cancer types. The order is based on hierarchical clustering. **d** The left heat map shows the proportion of differentially expressed pseudogenes in all pseudogenes and DDPs. The right panel represents the odds ratios in each cancer type. *P* < 0.01 except for COAD in two-sided Fisher’s exact tests

Motivated by this data, we extended our scope to cancer. We found that compared with ubiquitously expressed pseudogenes (20%), more cancer type-specific pseudogenes (35%) were DDPs (Fig. 7a, Additional file 1: Figs. S30-S31) (See Methods). These cancer type-specific pseudogenes might have specific functions and stand for novel elements unique to a certain type of cancer type [19]. Such type-specific pseudogene enrichment supported the notion that tissue development and tumorigenesis were intertwined [67]. Considering that some cancer type-specific pseudogenes might only represent biological features unique to a certain type of cancer type rather than play pivotal roles in cancerogenesis [20], we subsequently explored the differentially expressed pseudogenes between cancer samples and benign ones. A total of 173 to 2182 differentially expressed pseudogenes were identified from 17 cancer types, accounting for 9.2 to 32% of all differentially expressed (DE) genes (Fig. 7b), indicating the prominent contribution of pseudogenes to cancer transcriptome changes. Among these DE pseudogenes, more upregulated pseudogenes were detected than downregulated ones, and the upregulated ones have the potential to be used as therapeutic targets. An example of upregulated DDPs was *ABCC13*, which exhibited remarkably a higher expression level in colorectal cancer than in normal adjacent tissue (Additional file 1: Fig. S32). Interestingly, we also observed that the cancer types with identical organ origin showed similar pseudogene expression changes such as colon adenocarcinoma (COAD) and rectum adenocarcinoma (READ) (Fig. 7c). Such molecular features could be exploited to develop surrogate markers for cancer early screening and detection. We observed a high overlapping between DDPs and DE pseudogenes in 16 out of 17 cancer types (Fig. 7d). These results indicated that a rigorous investigation of dynamic pseudogenes identified in this study will provide informative insights into human disease and cancer biology in the future.

## Discussion

Much work has been done on non-coding RNAs, including long non-coding RNAs and circular RNAs [68–71], while the investigation on pseudogenes is still in its infancy. Here, we identified and characterized an expanded landscape of pseudogene transcription by adopting a hybrid sequencing method and integrating large-scale public transcriptome data. The unprecedented developmental stage span and depth enabled the sensitive detection of pseudogene transcription. The transcribed pseudogene proportion in our results far outnumbered that in current pseudogene databases (10% and 5% in human and mouse, respectively) [27, 35]. Such a difference might be attributed to strong tissue and stage specificities of pseudogenes, and the difference may increase with the growing number of samples and cell types studied. Likewise, we found that over 19% of pseudogenes could encode peptides, which eclipses previous reports. With the increased resolution, sensitivity, and sample size (including more tissues and developmental stages) in the future, MS technique could be used to systematically determine the translation ability and products of pseudogenes. Considering that some translated pseudogenes have been re-annotated as functional protein-coding genes (e.g., *PGK2*, *NANOGP8*, and *POU5F1B*) [18], we speculated that although not all translated pseudogenes carried functions, some of them might encode parent-independent functional proteins and contributed to phenotypic innovation.

Multiple evidence supported functions of pseudogenes including high conservation, robust expression, tissue and stage specificity, developmental dynamics, enriched TF binding, active regulation at promoters, proximity to m6A modification, frequent *trans* interactions, and involvement in cancer transcriptome changes (Additional file 8: Table S7). In addition, many pseudogenes regarded as functional candidates in this study have been previously experimentally verified to be related to important biological processes [72]. The unexpectedly high abundance of transcribed and translated pseudogenes we nominated highlighted that they might serve as a “gene or protein repository” and further investigations are needed to determine their effects on human biology and disease. We anticipate that the full-length PacBio sequencing data and sample-matched Illumina data of six major tissues in both sexes generated in this study would facilitate further functional studies.

Theoretical terms are the foundation for the development of scientific theories. Since the term “pseudogene” was introduced, this overhasty classification was assumed as “similar but defective” copies that were not able to be transcribed or translated. However, the emergence of high-throughput data and advanced experiment methods shed the light on the expression and function of pseudogenes, thus challenging the conventional opinion [18]. Moreover, unexpressed pseudogenes could contain cis-regulatory elements, and they might act as important regulators in human biology and health [1]. Therefore, one study has proposed the term “exapted pseudogene” to represent the functional renewal of a pseudogene and to eliminate this controversy. In this study, we put forward “awake paralogs” as an alternative description of expressed processed and unprocessed pseudogenes since the descriptive term should not make any functional inference without functional or biological validation. Meanwhile, the “asleep paralogs” (unexpressed pseudogenes) could serve as a “gene repository,” and they might wake up and confer fitness to the organism due to the changes in environment or genetic background in the future.

## Conclusions

Here, we analyzed the evolution and expression of mammalian pseudogenes within a developmental framework and provided a comprehensive expression profile of pseudogene transcriptomes by integrating PacBio long-read sequencing and large-scale RNA-seq data. We also identified the DDPs with enrichment of functional features, provided proof-of-principle evidence that DDPs contributed to organ development and might represent a new regulatory layer, and associated these DDPs with putative functions (i.e., metabolic process and DNA modification). Future studies combining experiments and emerging sequencing technologies will further uncover the regulatory profiles of these DDPs and elucidate their phenotypic consequences and underlying molecular mechanisms.

## Methods

### Pipeline for dating pseudogene age

The human and mouse pseudogene annotations, including their coordinates and biotypes (processed, unprocessed, unitary, and polymorphic pseudogene) generated and manually curated by GENCODE project [27], were downloaded from ENSEMBL compara [35]. We dated human and mouse pseudogenes based on synteny-based method [28]. First, we excluded the Y chromosome-located pseudogenes since the Y chromosome was largely invaded by transposable elements, and we also excluded the pseudogenes with more than 70% exonic regions covered by repeats. Afterwards, a total of 14,136 (92.7%) pseudogenes in human and 13,685 (96.8%) in mouse were retained for further analysis. We identified reciprocal best region of pseudogene’s each exon in other species based on whole genome alignment files generated by UCSC Genome Browser [73] and assigned phylogenetic distribution according to the most ancient exon following a parsimony rule. The divergence time was estimated from TimeTree [74]. Computational codes for dating age of pseuodgenes were uploaded to github. The parent coding genes of pseudogenes were obtained from Pseudogene.org [75]. Genome browser for *METTL21EP* was obtained from UCSC. For gene traffic analysis, we assumed that each chromosome would generate pseudogenes in proportion to the number of protein-coding genes on this chromosome and that chromosomes received pseudogenes in proportion to the size of the chromosome. We then calculated the difference between observed and expected number of pseudogenes on autosomes and X chromosome, and we conducted statistics analysis using Fisher’s exact test.

### Illumina sample preparation

Total RNAs from mouse (C57BL/6J) adult brain, cerebellum, heart, colon, and gonad in both sexes were extracted and were subsequently treated with Ribo-off rRNA Depletion Kit to remove ribosome RNA (rRNA). Then the VAHTS TM Stranded mRNA-seq Library Prep Kit for Illumina was used for strand-specific library construction. Afterwards, the library was sequenced on the Illumina Nova platform.

### Expression analysis

Developmental transcriptome data of human and mouse were downloaded from EBI ArrayExpress under accession number E-MTAB-6814 and E-MTAB-6798, respectively, which covered the developmental stages from organogenesis to adulthood including six major tissues (brain, heart, cerebellum, liver, kidney, and gonad in both sexes) [25]. For human data, we applied STAR (v2.6.1) [76], which is accurate in distinguishing similar paralogs [21] (Additional file 1: Figs. S33-S35), for mapping reads and featureCounts (v2.0.0) for quantifying mapped reads, respectively [77]. We combined our PacBio annotation with ENSEMBL reference annotation to quantify mouse data. Considering the high sequence similarity between pseudogenes and their parent coding cognates, we only retained uniquely mapped reads for quantification to achieve an unbiased analysis. We determined the gene expression level using FPKM (reads per kilobase million), and introduced tau value to estimate the tissue specificity of genes [78]. The tau value was calculated in the following formula:

$$\textrm{tau}=\frac{\sum_{i=1}^n\left(1-{y}_i\right)}{n-1};{y}_i=\frac{x_i}{\underset{1\le i\le n}{\max}\left({x}_i\right)}$$

where *x*_*i*_ indicates the expression level of gene *x* in tissue *i*. Likewise, the same calculation formula was applied to developmental stage specificity. Both and tissue and stage specificity tau values ranged from 0 (broad expression) to 1 (highly specific expression).

### PacBio sequencing

The RNAs were extracted from adult mouse brain, cerebellum, heart, colon, and gonad in both sexes, and somatic tissues were pooled into one sample in each sex. The samples were used to prepare cDNA library using P Clontech SMARTer PCR cDNA Synthesis Kit and PrimeSTAR GXL DNA Polymerase, followed by library construction by using SMRTbellTM Template prep and sequencing on the Pacific Biosciences Sequel I platform. Specifically, we sequenced high-quality RNA from somatic and gonad tissues of two sexes of the mouse on a Sequel I platform, including 4 RNA libraries (6 Pacbio flow-cells), and produced 1,946,228 raw reads.

### PacBio data processing

The raw data were subjected to SmrtLink Pipeline ccs (v5.0.0) for self-correction to obtain full-length circular consensus sequencing (CCS) reads based on PacBio recommended pipeline. We applied the SmrtLink Pipeline Cluster subprogram to cluster the full-length non-chimeric sequences into full-length transcripts (longer than 300bp) and retained 899,237 full-length transcripts supported by at least one full-length non-chimeric sequence through Isoseq 3 [79]. The LoRDEC program (v0.7) [80] was leveraged to correct the full-length transcripts based on the results of Illumina RNA-seq to improve the accuracy of the third generation transcripts. After being corrected by LoRDEC using next-generation RNA-seq data, the third generation transcripts were mapped to reference genome (Ensembl 98) through minimap2 (version, 2.17-r954-dirty) [81]. Then the redundant transcripts model (GFF3 format) for each high-quality non-chimeric CCS read from minimap2 output were collapsed using the Python script (collapse_isoforms_by_sam.py) from cDNA_cupcake ((https://github.com/Magdoll/cDNA_Cupcake) to generate a non-redundant set of transcript model (termed as Iso set). Gffcompare (GFF Utilities, v0.11.2) [82] was used to compare each isoform in Iso set with existing mouse Ensembl gene models (termed as reference set). Among the 49,914 full-length transcripts, 332 were derived from pseudogene loci. The genes annotated in the reference set but not overlapped with Iso-seq annotation at the same strand were merged with Iso-seq annotation to form the final annotation. The pseudogene transcripts were identified and classified as those assigned the name of reference pseudogenes by Gffcompare.

### Identification and characterization of DDPs

In each tissue, DDPs were identified using an R package masigPro, which was designed for time-course transcriptome data [43]. In general, the CPM (counts per million) was used to calculate a goodness-of-fit (R^2^) metric. We ran maSigPro using the log-transformed time after conception with a degree = 3. A pseudogene with R^2^ >0.3 in a tissue was defined as DDP in this tissue. The pseudogenes exhibiting developmentally dynamics in at least one tissue were finally classified as DDPs. The lists of DDPs in each tissue in human and mouse are provided in Additional file 9: Table S8, Additional file 10: Table S9, Additional file 11: Table S10, Additional file 12: Table S11, Additional file 13: Table S12, Additional file 14: Table S13 and Additional file 15: Table S14. DESeq2 (v1.30.1) was applied for differential expression analysis [83].

We characterized the DDPs using different metrics. The length of pseudogenes and parent coding genes was measured through non-redundent exonic regions. Promoter regions were defined as the regions 2kb upstream to 1kb downstream of the transcription start sites (TSSs). To enable comparison between protein-coding genes and pseudogenes, their TSSs were defined as the starting coordinate of the first exon of each gene. The intergenic regions (matched length, 3kb) were randomly selected as negative control. The transcription factor (TF) binding information was retrieved from GTRD, a database collecting more than 5000 ChIP-seq experiment data of human and mouse TFs [84]. Bedtools intersect was utilized to detect the overlapping of TF binding sites and promoter regions [85]. Diversity of TFs in a region was defined as the number of types of different TFs. Number and diversity of TFs were used for evaluating transcriptional complexity.

Epigenetic data from Roadmap Epigenomics Project were obtained from the data portal (http://egg2.wustl.edu/roadmap/). A total of 127 consolidated epigenomes were included in this work. A promoter with more than 1 base pair overlapping with a chromHMM state was defined as annotated by this state. The whole genome sequences were plotted as background. We intersected the gene body regions with H3k27ac and DHS (DNase hypersensitivity) data. The distribution profile of H3K27ac and DHS peaks relative to transcription start site (TSS) was generated by deepTools [86]. The random regions were acquired from randomly shuffled intergenic regions without any overlapping with known gene regions. The N6-methyladenosine methylome data were obtained from REPIC, a publicly available dataset with 10 million peaks called from m6A-seq and MeRIP-seq data [87]. We retrieved RNA-binding proteins (RBPs) data of human and mouse from oRNAment [88]. Post-transcription complexity was determined based on the number and enrichment of m6A and RBPs. For RIC-seq (RNA in situ conformation sequencing) data, we calculated the interaction pairs including mRNA-mRNA, mRNA-dynamic pseudogene, and mRNA-non-dynamic pseudogene. Poly(A) signals were obtained from PolyASite [89]. GO analysis was performed using clusterProfiler [90].

### Identification of disease- and cancer-related pseudogenes

A detailed list of GWAS SNPs was obtained from the National Human Genome Research Institute’s (NHGRI) GWAS catalog [66]. And unique SNPs were retained for further analysis. Gene expression datasets were generated by TCGA project for cancer-associated analysis. A total of 33 cancer types were included in this work. Gene expression profile (raw read count files) was downloaded by the R package TCGAbiolinks [91]. Next, we excluded the protein-coding genes and pseudogenes with zero read in all samples. The genes defined as cancer type-specific needed to meet two criteria: (1) the ratio of the gene expression level in a certain cancer type to the sum of that in all 33 cancer types was more than 15%; (2) the ratio of gene expression level in any of the rest 32 cancer types to the sum of that in all 33 cancer types was less than 5%. In contrast, the genes defined as ubiquitous pseudogenes met two criteria: (1) the ratio of the gene expression level in a certain cancer type to the sum of that in all 33 cancer types was more than less than 30%, and (2) the ratio of gene expression level in each of top 5 cancer types to the sum of that in all 33 cancer types was more than 5%. To avoid the dominance of tumor or normal tissues in differential expression analysis by DESeq2, only 17 cancer types with more than 5 normal and 5 tumor samples were retained. Colorectal cancer and adjacent samples for IGV were obtained from Li et al. [92].

### Coding potential assessment of pseudogene transcripts

The coding potential of pseudogenes was assessed by using CPC2 (0.1) [62] and CPAT (v.1.2.4) [63]. CPC2 is a fast coding potential calculator based on sequence intrinsic features. CPAT uses an alignment-free logistic regression model to recognize coding potential based on sequence features, and recommended cutoff (> 0.364) was used to identify the pseudogene transcripts with coding potential. We downloaded Ribo-seq data and ORF information from RPFdb [93] collecting the most comprehensive ribosome-profiling data to verify the coding potential of all candidates. The maximum FPKM of a given gene among all Ribo-seq samples was defined as its FPKM value and a pseudogene candidate with FPKM >1 was validated as a translated one.

## Supplementary Information

Additional file 1: Figure S1. Number of processed pseudogenes with different evolutionary ages. Figure S2. Expression pattern of Iso-seq detected pseudogene *Gm13857* and *4632415L05Rik*. Figure S3. The distribution of expression correlation coefficient between pseudogenes and parent coding genes. Figure S4. The distribution of expression correlation coefficient between expressed pseudogenes and parent coding genes. Figure S5. The expression level between pseudogenes and parent coding genes. Figure S6. Principle component analysis (PCA) based on mouse pseudogenes using developmental transcriptome data. Figure S7. Principle component analysis (PCA) based on mouse pseudogenes using our RNA-seq data. Figure S8. Percentage of sex-biased and unbiased pseudogene, lncRNA, and protein-coding gene in each tissue. Figure S9. PCA on the 1:1 orthologous protein-coding genes between human and mouse. Figure S10. Fraction of transcribed pseudogenes with different origination mechanisms under a range of FPKM cutoffs in human and mouse. Figure S11. Expression level ratio between pseudogene parent coding genes and non-pseudogene-generating coding genes. Figure S12. Tissue specificity of mouse pseudogene expression. Figure S13. Distribution of the organ in which maximum expression is observed for mouse pseudogenes. Figure S14. Heatmap for human pseudogenes expression using a dataset covering 32 human adult tissues. Figure S15. Heatmap for mouse pseudogenes expression using ENCODE data. Figure S16. Number of pseudogenes show higher expression level in each tissues. Figure S17. Developmental stage-specificity of pseudogene expression in mouse. Figure S18. The distribution of expression correlation coefficient between expressed pseudogenes and parent coding genes. Figure S19. Distribution of transcript length for dynamic and non-dynamic mouse pseudogenes. Figure S20. Distribution of transcript length for parent coding genes of dynamic and non-dynamic pseudogenes. Figure S21. Number and types of TFs overlapping the promoters of protein-coding genes, dynamic pseudogenes, non-dynamic pseudogenes, and randomly shuffled intergenic regions in human genome. Figure S22. Diversity of TFs overlapping the promoters of protein-coding genes, Iso-seq detected dynamic pseudogenes, dynamic pseudogenes, non-dynamic pseudogenes, and randomly shuffled intergenic regions in mouse genome. Figure S23. Poly(A) signal and transcription factors binding sites of a genomic locus *1700031M16Rik*. Figure S24. Proportion of state annotated overlapped with promoter of each type of genes annotated with each epigenetic state, summed across all epigenomes. Figure S25. Conservation score of dynamic and nondynamic pseudogenes. Figure S26. Fraction of dynamic loci for human and mouse pseudogenes of different evolutionary ages. Figure S27. Number of co-expressed pair of dynamic and non-dynamic pseudogenes. Figure S28. Coverage plot and raw alignments from Ribo-seq reads (SRR837789) for a process pseudogene *AL589987.1* (chrX:140,091,874-140,092,692). Figure S29. Coverage plot and raw alignments from Ribo-seq reads (SRR837789) for a process pseudogene *NDUFB4P8* (chr1:1,378,666-1,379,032). Figure S30. Expression pattern of cancer type-specific pseudogenes across 32 cancer types. Figure S31. Expression pattern of ubiquitously expressed pseudogenes. Figure S32. Expression level of *ABCC13* in colorectal cancer (CRC) and adjacent tissues. Figure S33. Distribution of sequence identity between pseudogene and parent gene pairs. Figure S34. Identity of reads derived from pseudogene loci. Figure S35. Number of mismatch of reads derived from *4632415L05Rik*.

Additional file 2: Table S1. The generation and enter of pseudogenes on X chromosome and autosomes.

Additional file 3: Table S2. The list for pseudogenes that were detected by PacBio sequencing.

Additional file 4: Table S3. Co-expression pair between pseudogenes and coding genes.

Additional file 5: Table S4. GO enrichment for coding genes co-expressed with dynamic pseudogenes.

Additional file 6: Table S5. GO enrichment for coding genes co-expressed with non-dynamic pseudogenes.

Additional file 7: Table S6. The detailed information of pseudogene ORF.

Additional file 8: Table S7. Number of expressed pseudogenes, Iso-seq detected pseudogenes, and DDPs.

Additional file 9: Table S8. DDPs among different tissues in human.

Additional file 10: Table S9. DDPs among different tissues in mouse.

Additional file 11: Table S10. Expression level of human DDPs in developmental transcriptome.

Additional file 12: Table S11. Expression level of human DDPs in GTEx project.

Additional file 13: Table S12. Expression level of human DDPs in a dataset of 32 human adult tissues.

Additional file 14: Table S13. Expression level of mouse DDPs in developmental transcriptome.

Additional file 15: Table S14. Expression level of mouse DDPs in ENCODE project.

Additional file 16. Review history.

## Data Availability

All PacBio and RNA-seq data generated by this study is deposited in the Gene Expression Omnibus (GEO) under accession GSE176018 [94]. The public data used in this article were downloaded from the original published papers. Developmental transcriptome for human (E-MTAB-6814) and mouse (E-MTAB-6798) were downloaded from EMBL-EBI [95]. RNA-seq data of 32 adult human tissues were downloaded from EMBL-EBI (E-MTAB-2836) [96]. RNA-seq of adult mouse tissues generated by ENCODE were downloaded from EMBL-EBI (E-GEOD-36025) [97]. All scripts used in this manuscript have been deposited in Github repository (GNU General Public Licence v3.0) [98] and Zenodo [99].
