# Supplementary material for: Evolution and function of developmentally dynamic pseudogenes in mammals
Source: Genome Biol. 2022 Nov 8;23:235. doi: 10.1186/s13059-022-02802-y (PMC9641868; doi:10.1186/s13059-022-02802-y)
Supplement: Supplementary file 1 — Additional file 1: Figure S1. Number of processed pseudogenes with different evolutionary ages. Figure S2. Expression pattern of Iso-seq detected pseudogene Gm13857 and 4632415L05Rik. Figure S3. The distribution of expression correlation coefficient between pseudogenes and parent coding genes. Figure S4. The distribution of expression correlation coefficient between expressed pseudogenes and parent coding genes. Figure S5. The expression level between pseudogenes and parent coding genes. Figure S6. Principle component analysis (PCA) based on mouse pseudogenes using developmental transcriptome data. Figure S7. Principle component analysis (PCA) based on mouse pseudogenes using our RNA-seq data. Figure S8. Percentage of sex-biased and unbiased pseudogene, lncRNA, and protein-coding gene in each tissue. Figure S9. PCA on the 1:1 orthologous protein-coding genes between human and mouse. Figure S10. Fraction of transcribed pseudogenes with different origination mechanisms under a range of FPKM cutoffs in human and mouse. Figure S11. Expression level ratio between pseudogene parent coding genes and non-pseudogene-generating coding genes. Figure S12. Tissue specificity of mouse pseudogene expression. Figure S13. Distribution of the organ in which maximum expression is observed for mouse pseudogenes. Figure S14. Heatmap for human pseudogenes expression using a dataset covering 32 human adult tissues. Figure S15. Heatmap for mouse pseudogenes expression using ENCODE data. Figure S16. Number of pseudogenes show higher expression level in each tissues. Figure S17. Developmental stage-specificity of pseudogene expression in mouse. Figure S18. The distribution of expression correlation coefficient between expressed pseudogenes and parent coding genes. Figure S19. Distribution of transcript length for dynamic and non-dynamic mouse pseudogenes. Figure S20. Distribution of transcript length for parent coding genes of dynamic and non-dynamic pseudogenes. Figure S21. Number and types [file 13059_2022_2802_MOESM1_ESM.docx]

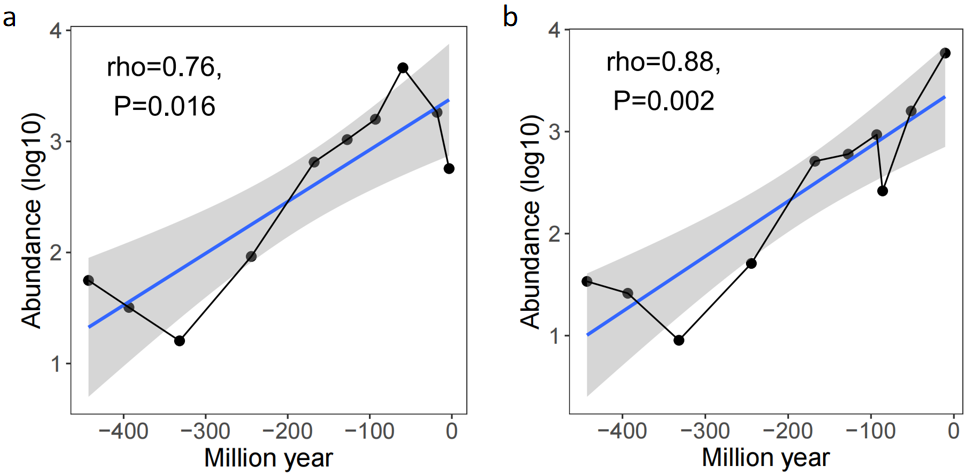


Figure S1. Number of processed pseudogenes with different evolutionary ages. (a) for human; (b) for mouse.


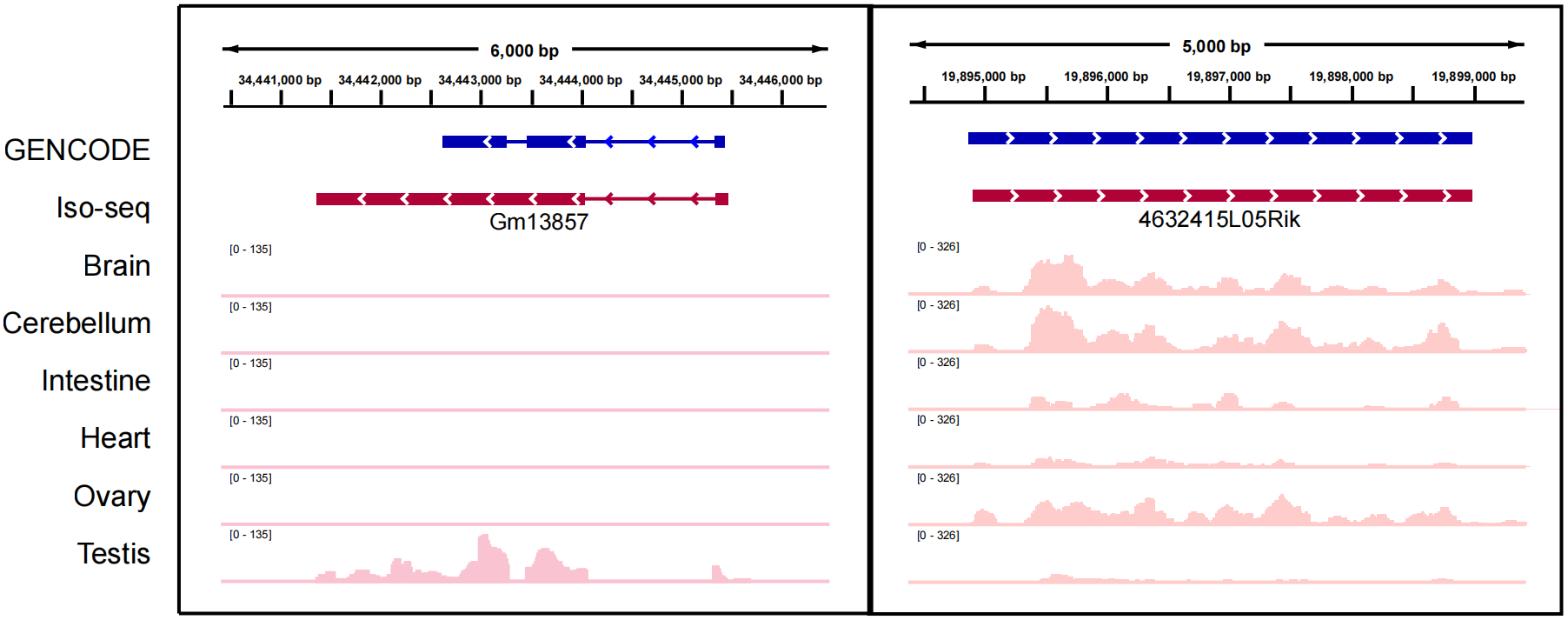


Figure S2. Expression pattern of Iso-seq detected pseudogene *Gm13857* and *4632415L05Rik*.


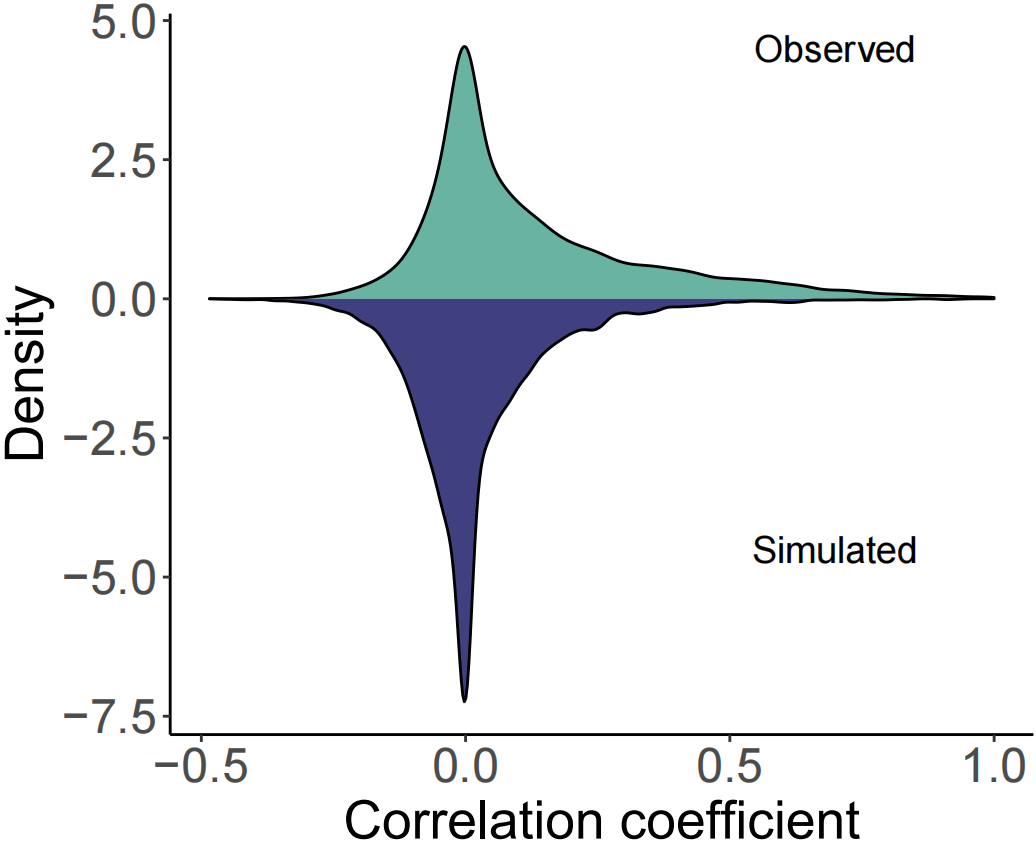


Figure S3. The distribution of expression correlation coefficient between pseudogenes and parent coding genes. The panel above 0 is observed values and below 0 is simulated values. The median observed positive and negative correlation coefficient (R) is 0.12 and -0.05, respectively. The median simulated positive and negative R is 0.06 and -0.06, respectively.


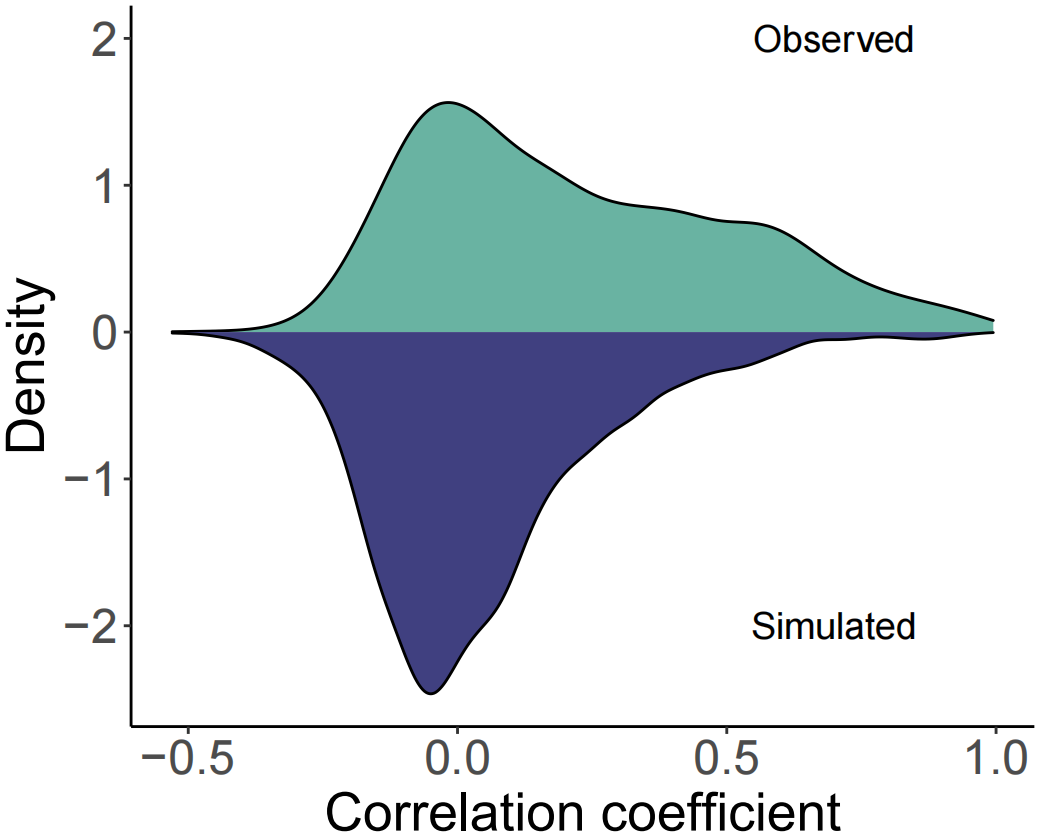


Figure S4. The distribution of expression correlation coefficient between expressed pseudogenes and parent coding genes. The panel above 0 is observed values and below 0 is simulated values. The median observed positive and negative correlation coefficient (R) is 0.31 and -0.08, respectively. The median simulated positive and negative R is 0.15 and -0.09, respectively.


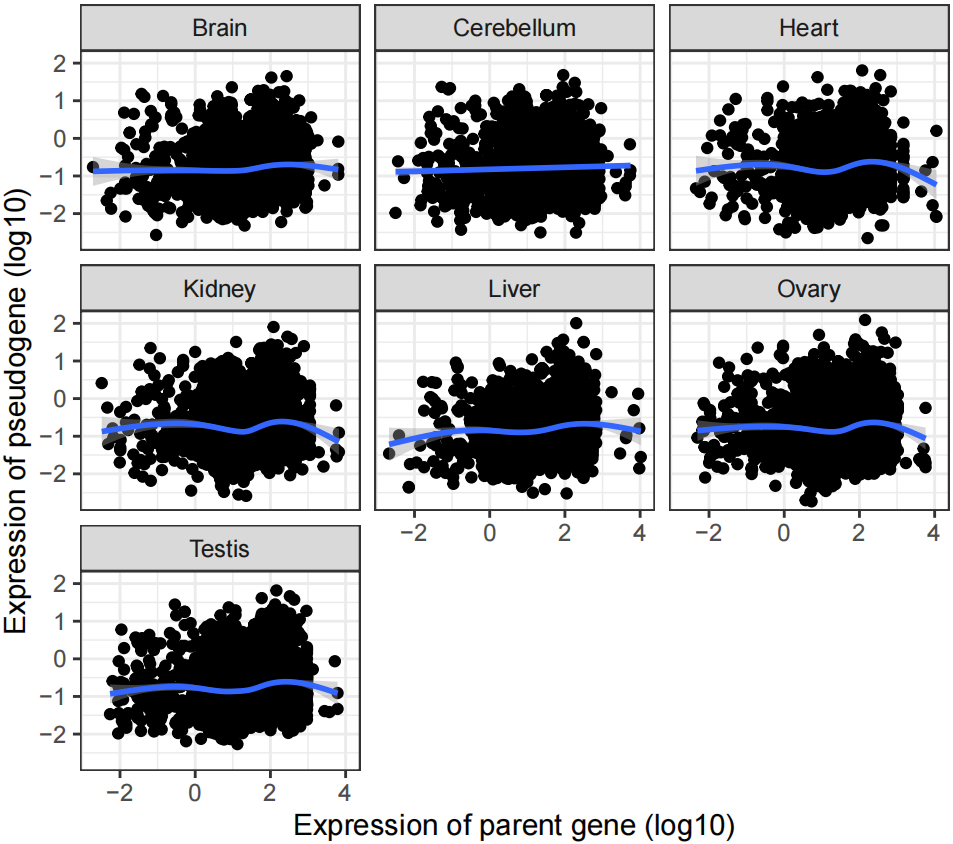


Figure S5. The expression level between pseudogenes and parent coding genes. Each point indicates a gene pair.


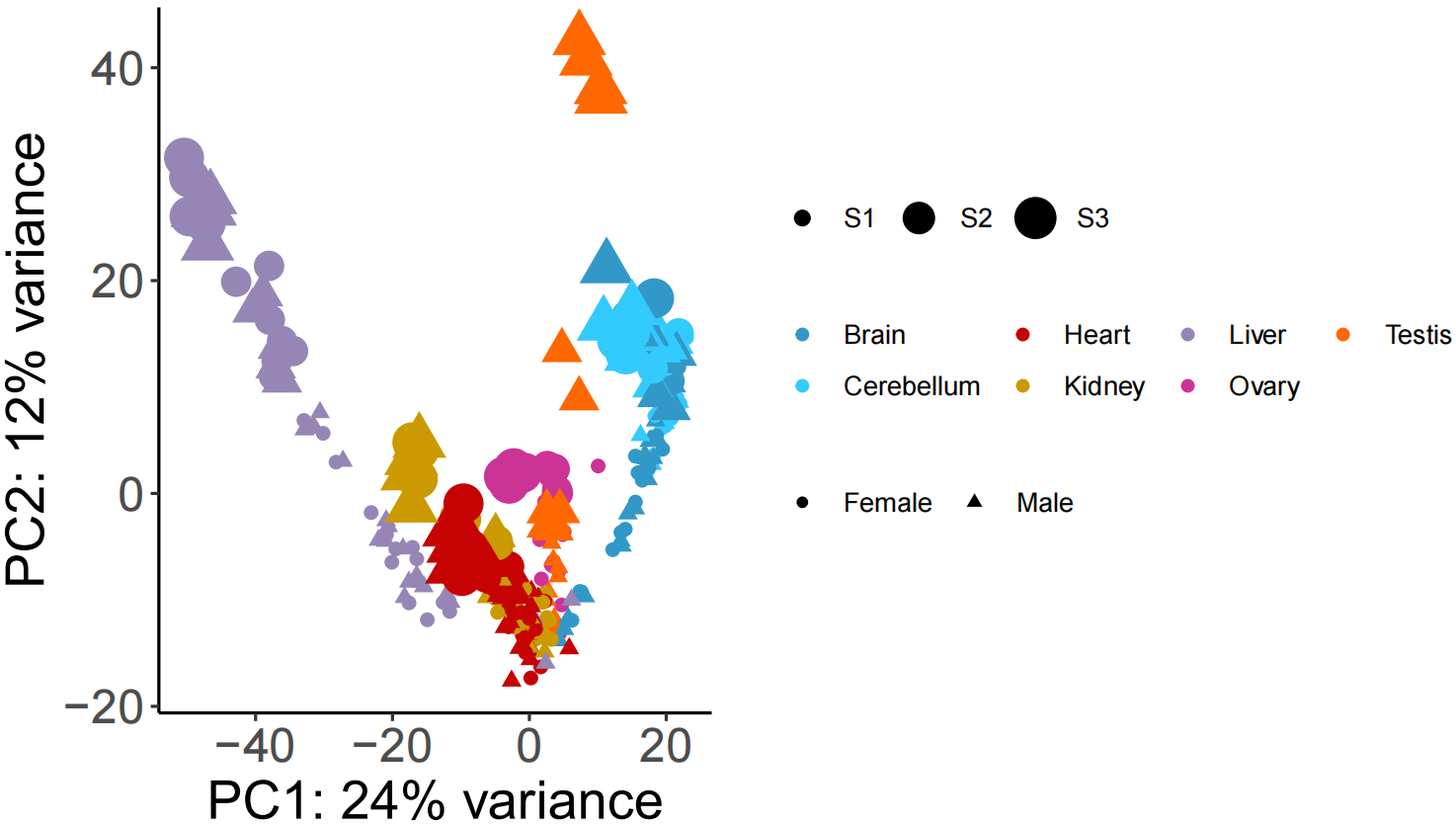


Figure S6. Principle component analysis (PCA) based on mouse pseudogenes using developmental transcriptome data.


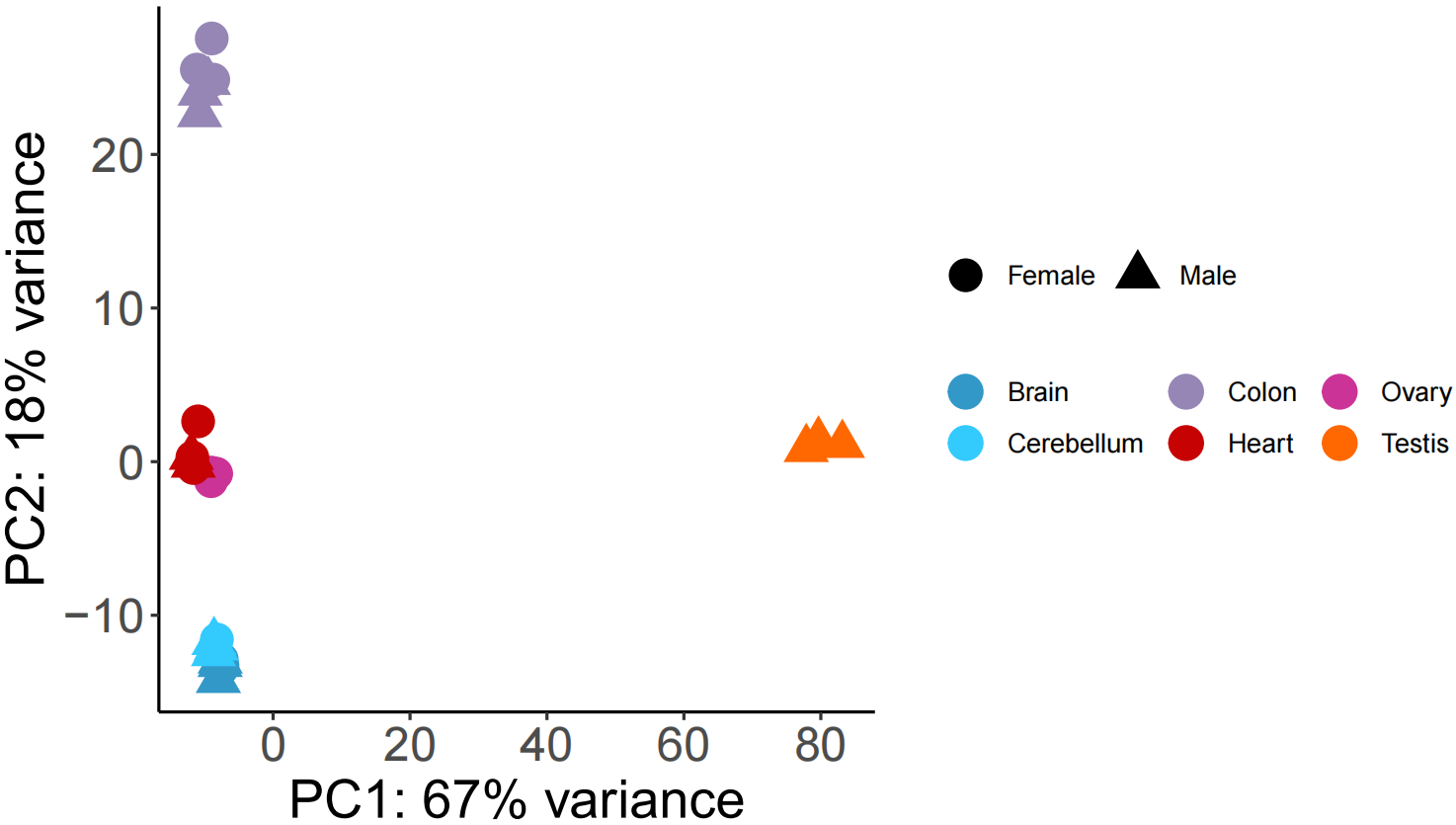


Figure S7. Principle component analysis (PCA) based on mouse pseudogenes using our RNA-seq data.


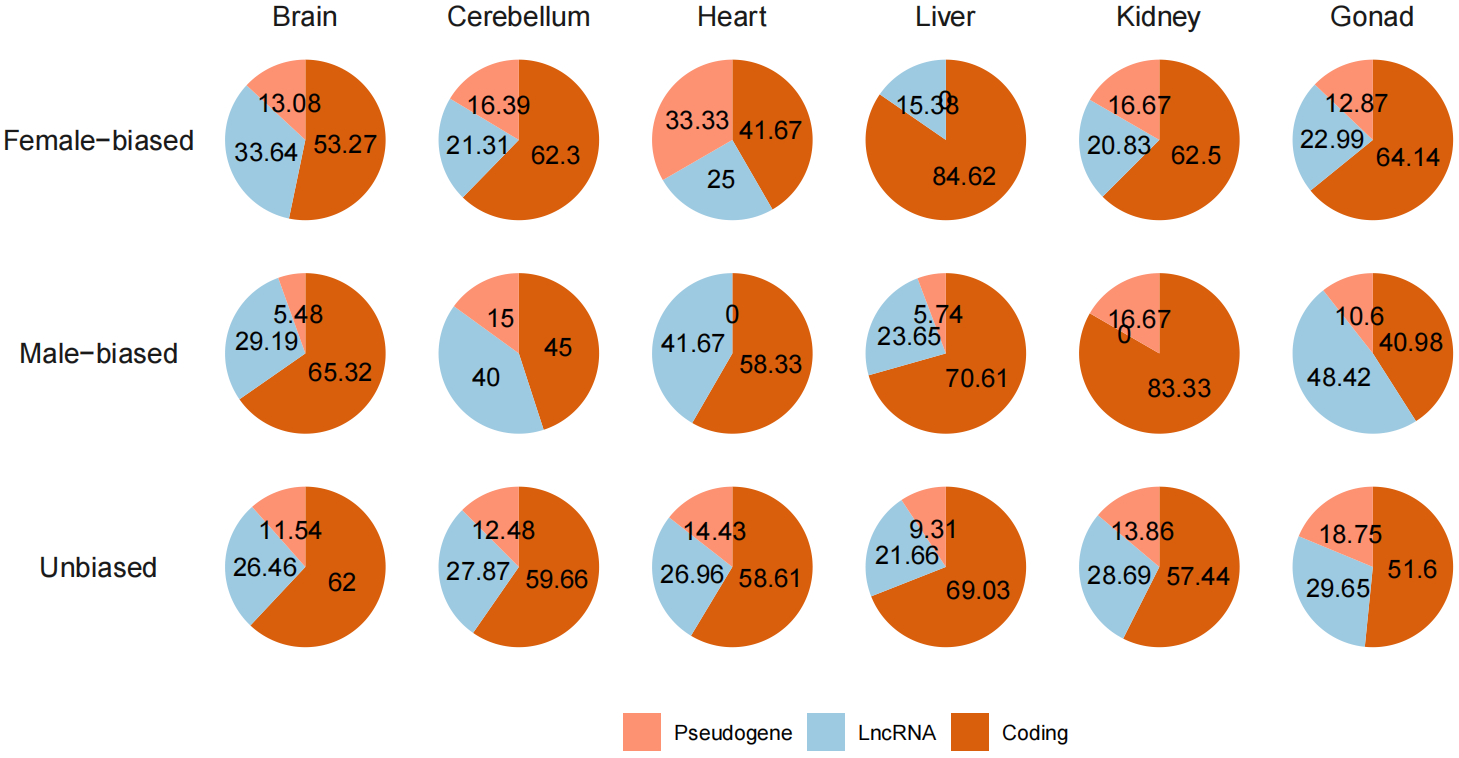


Figure S8. Percentage of sex-biased and unbiased pseudogene, lncRNA, and protein-coding gene in each tissue.


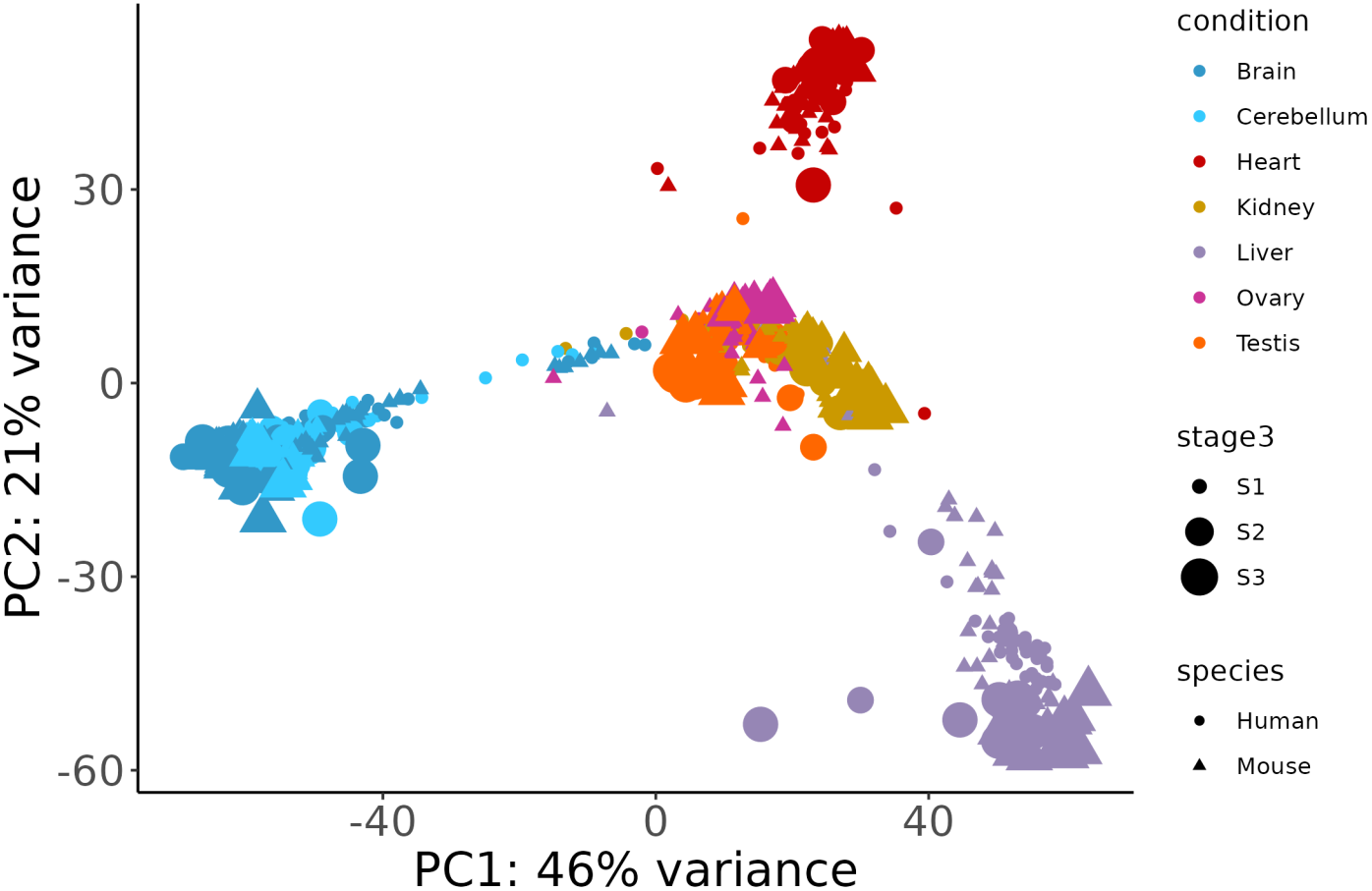


Figure S9. PCA on the 1:1 orthologous protein-coding genes between human and mouse.


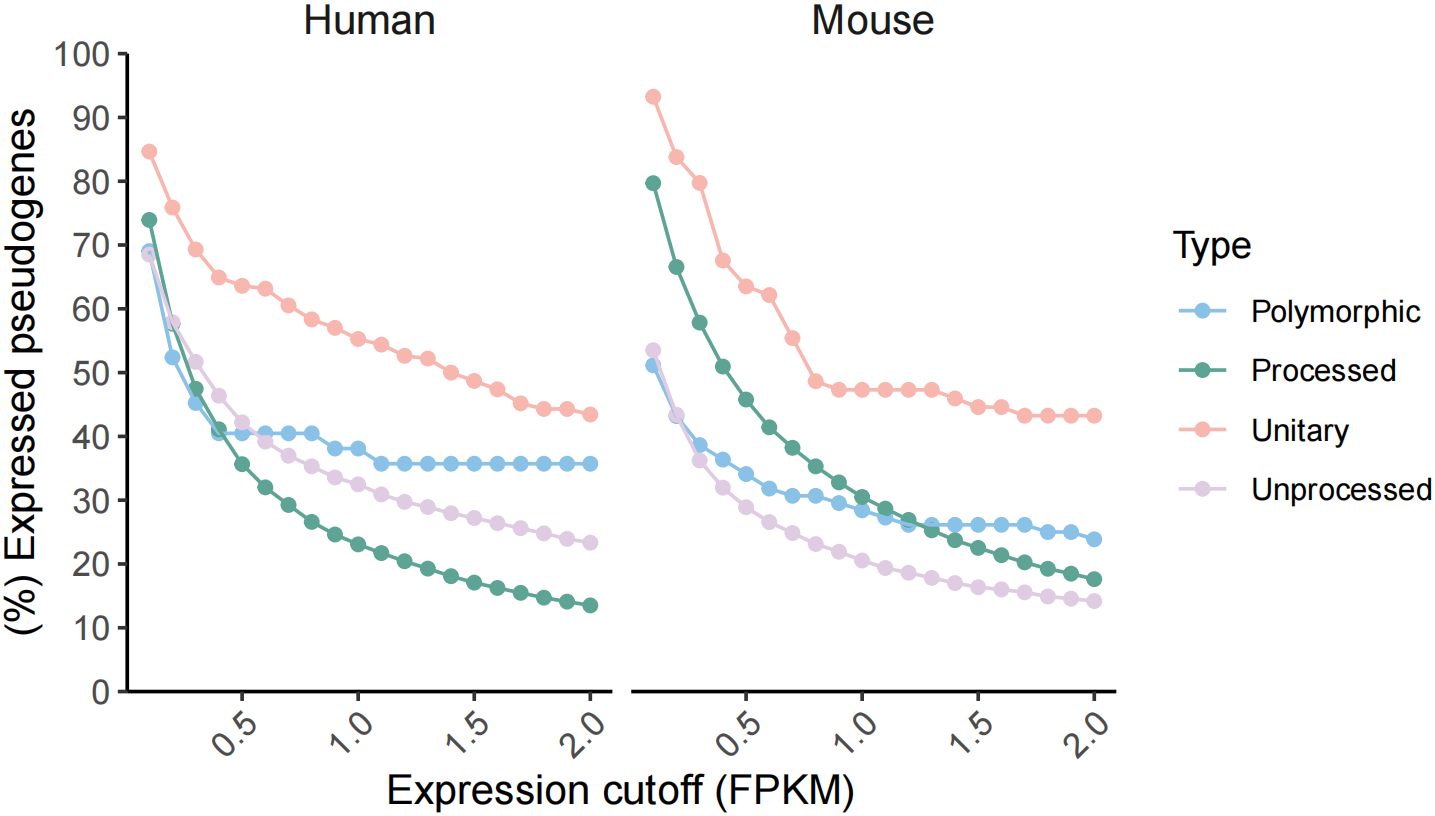


Figure S10. Fraction of transcribed pseudogenes with differnt origination mechanisms under a range of FPKM cutoffs in human and mouse.


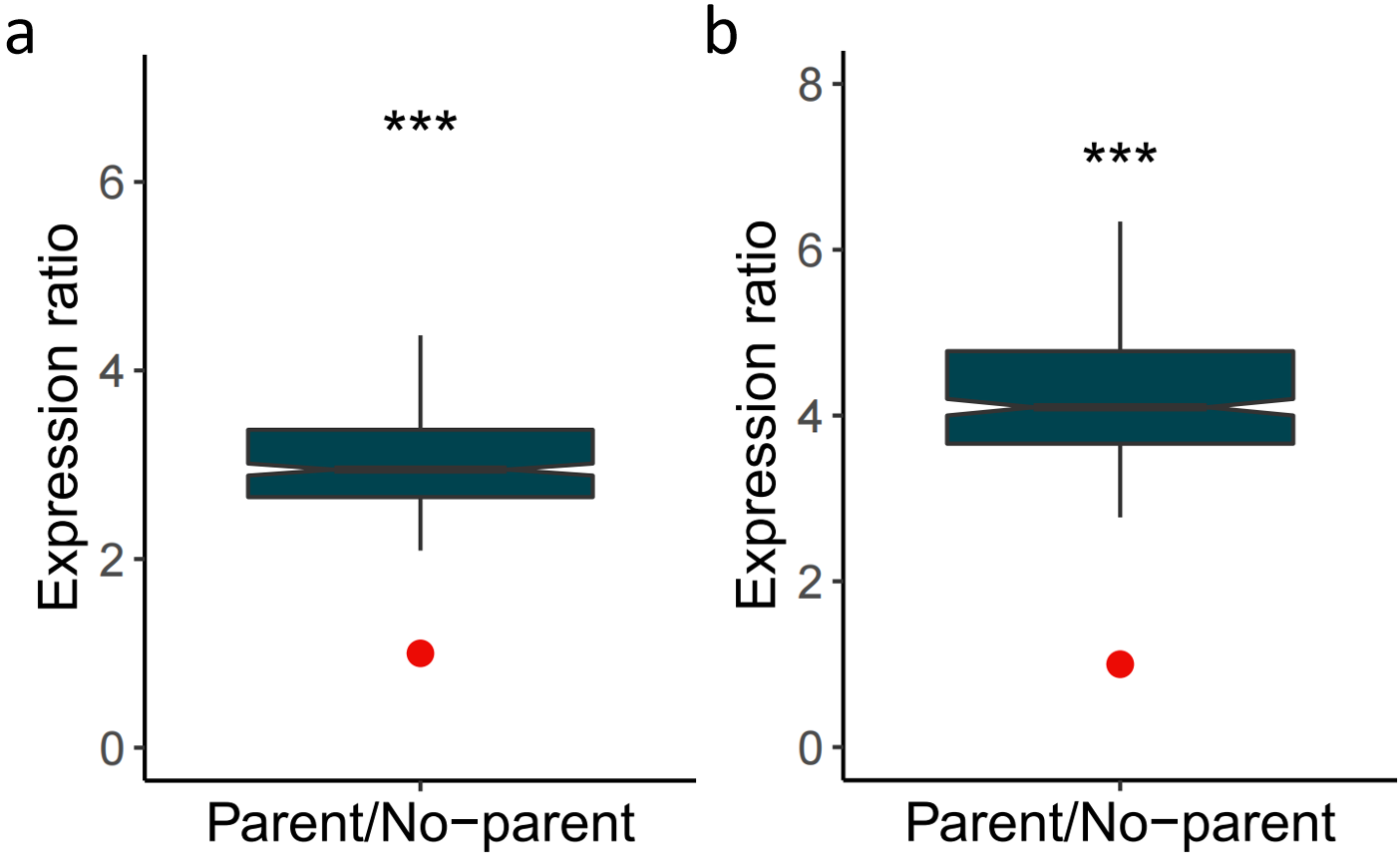


Figure S11. Expression level ratio between pseudogene parent coding genes and non-pseudogene-generating coding genes. In each sample, we separatly calculated the expression level of pseudogene-generating coding genes and non-pseudogene-generating coding genes. Then we computed the ratio between them. Red point represents the ratio =1 under the assumption of equal expression level between the two types of coding genes. (a) for all pseudogenes, (b) only for processed pseudogenes.


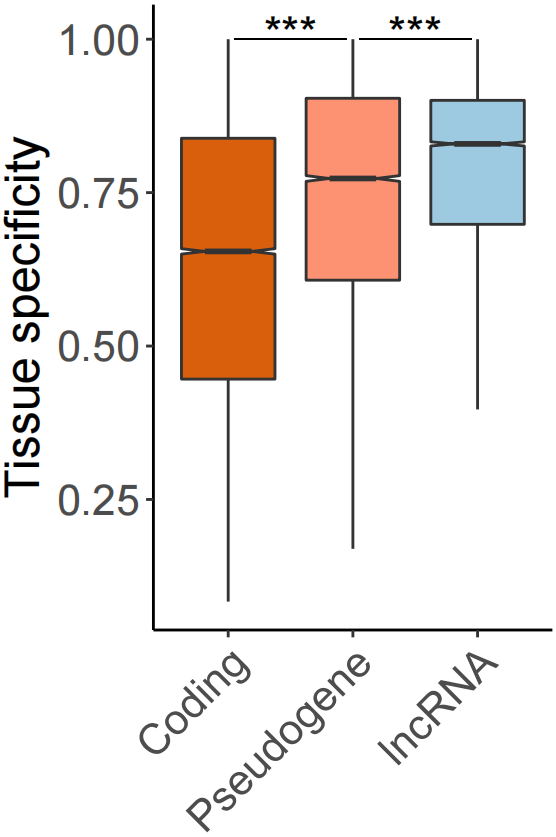


Figure S12. Tissue specificity of mouse pseudogene expression.


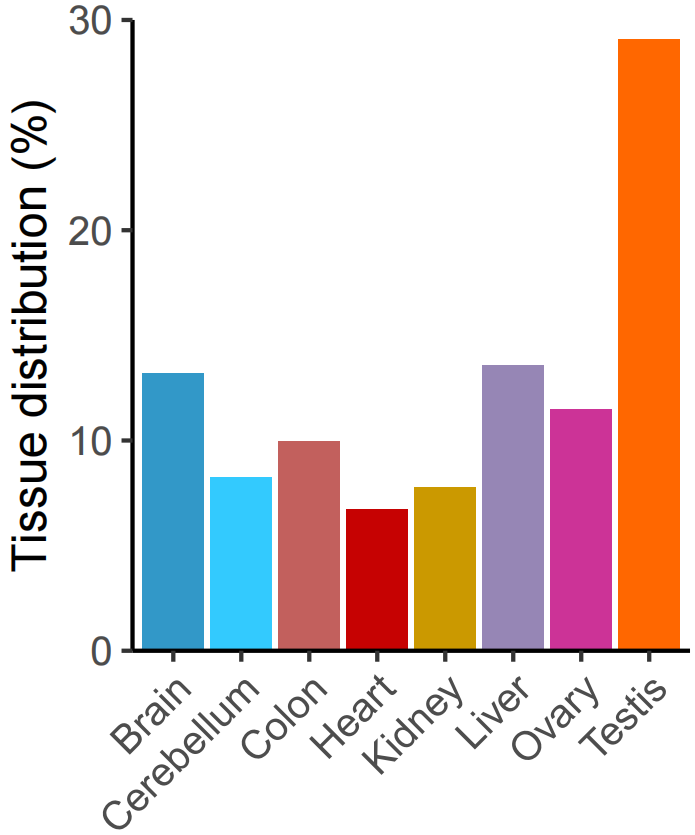


Figure S13. Distribution of the organ in which maximum expression is observed for mouse pseudogenes.


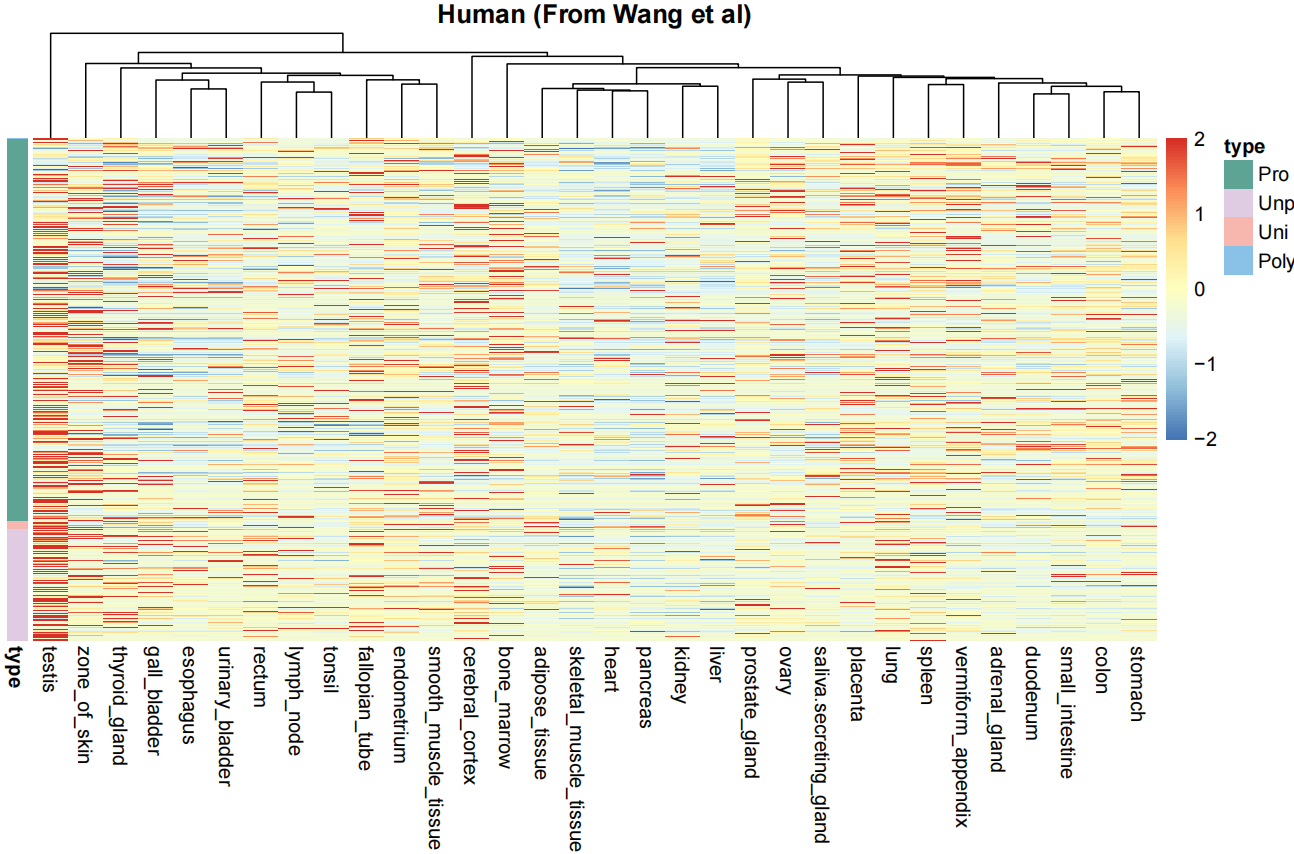


Figure S14. Heatmap for human pseudogenes expression using a dataset covering 32 human adult tissues.


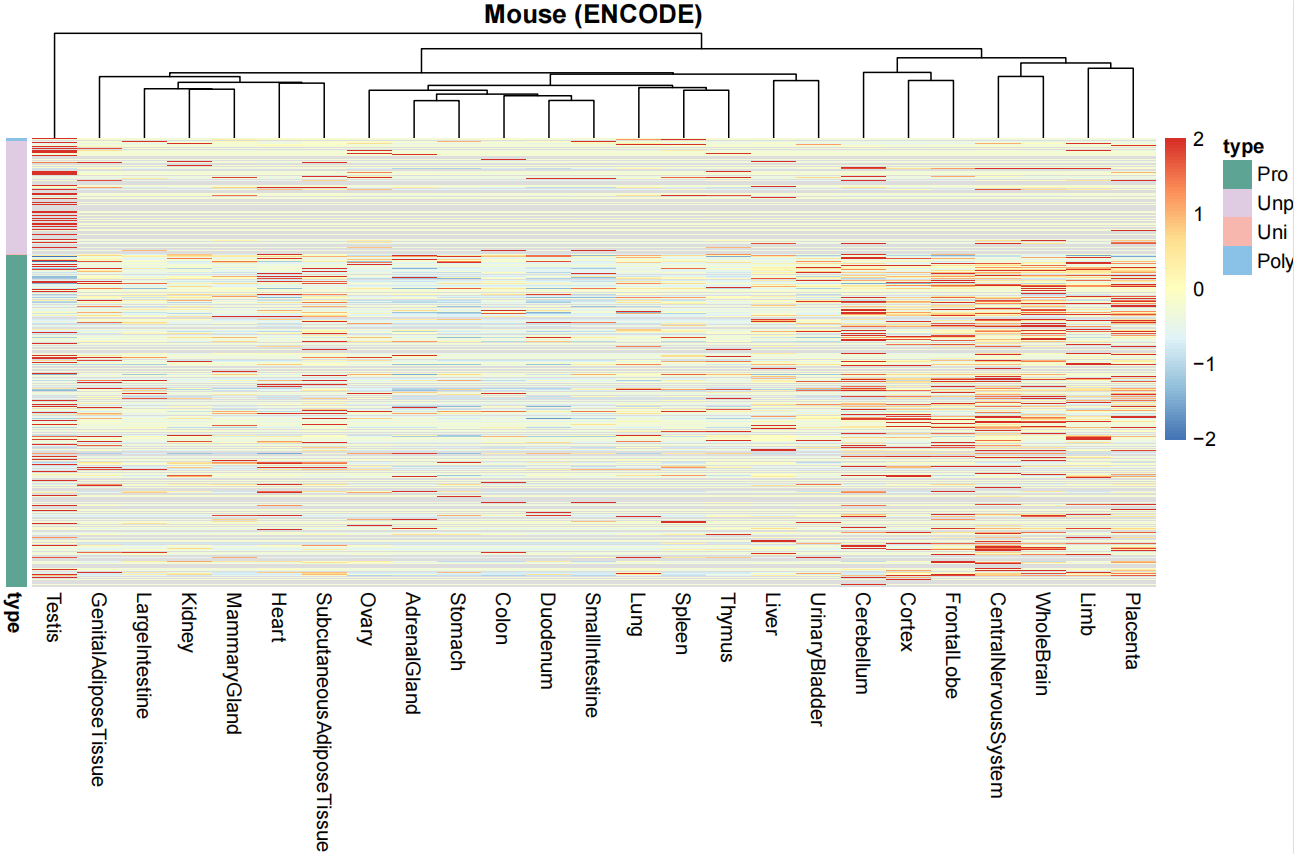


Figure S15. Heatmap for mouse pseudogenes expression using ENCODE data.


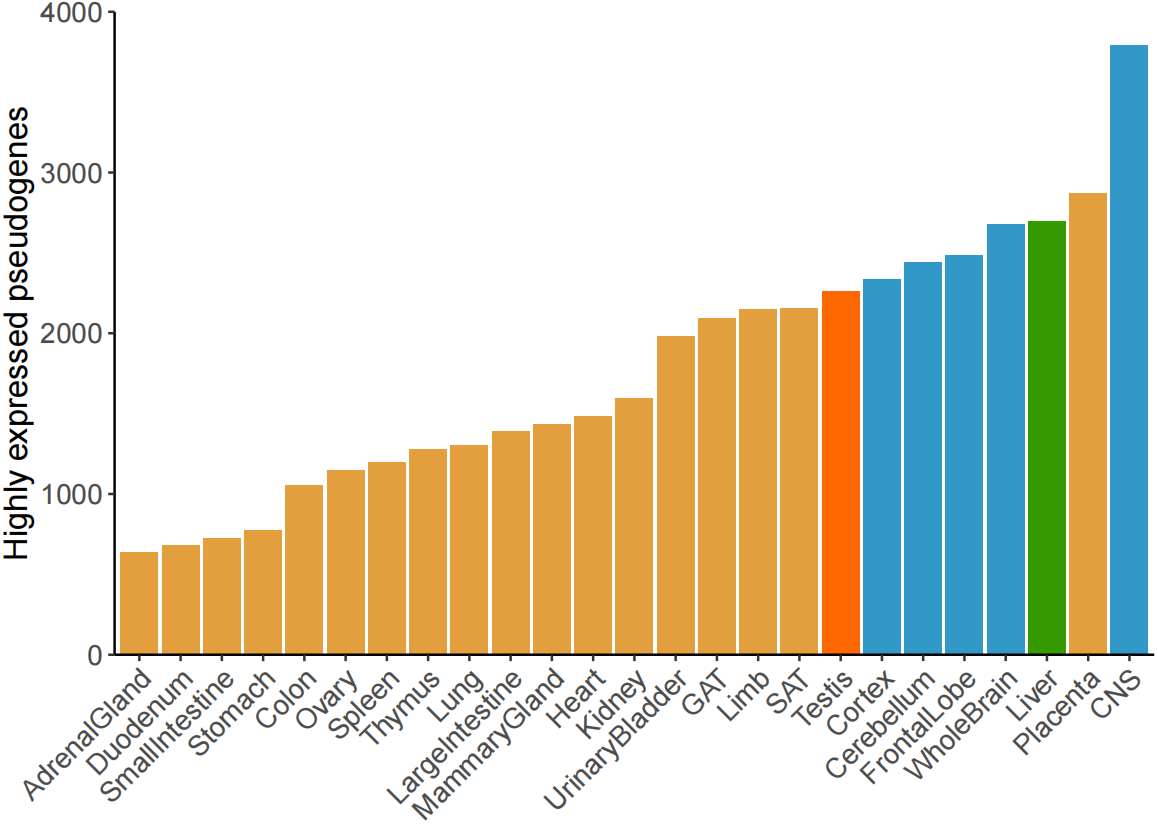


Figure S16. Number of pseudogenes show higher expression level in each tissues.


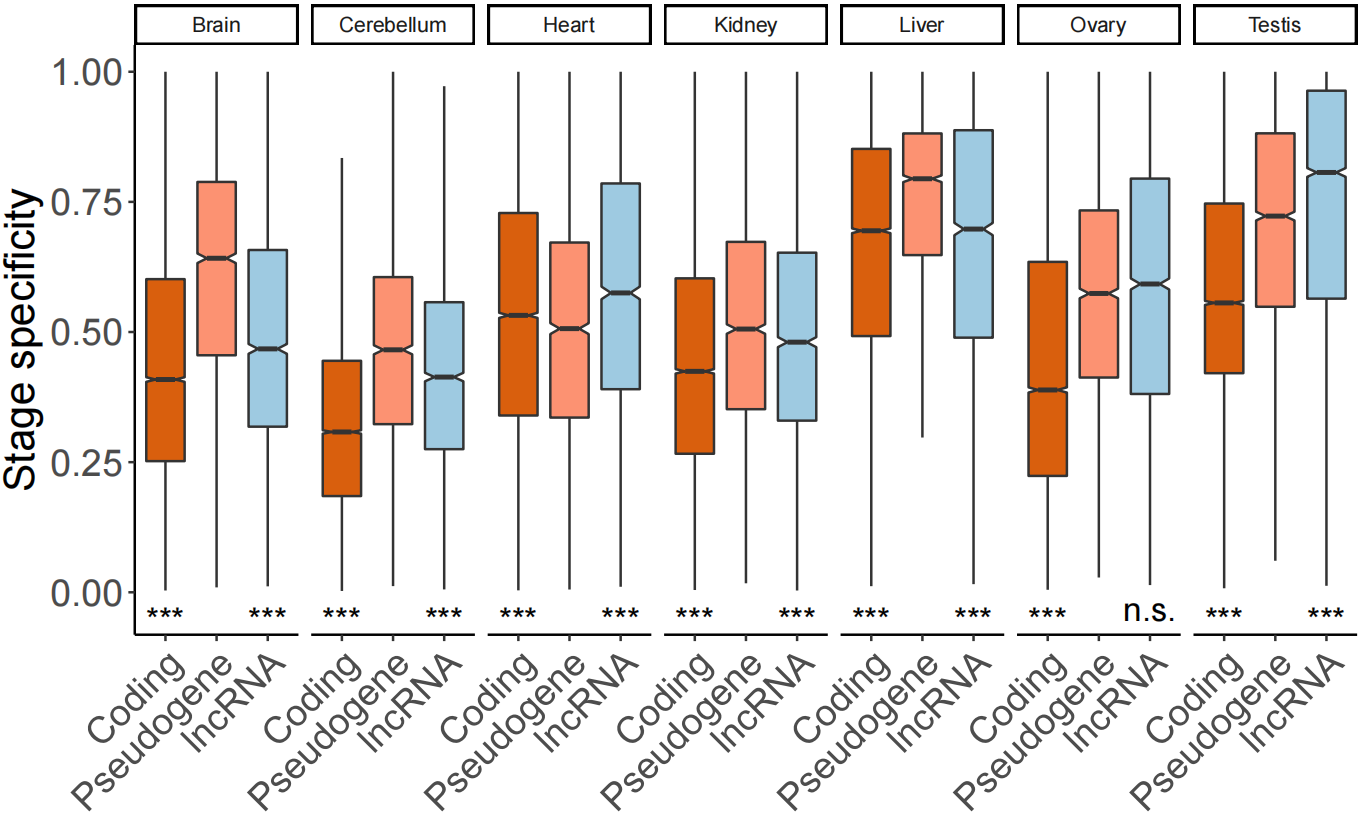


Figure S17. Developmental stage-specificity of pseudogene expression in mouse.


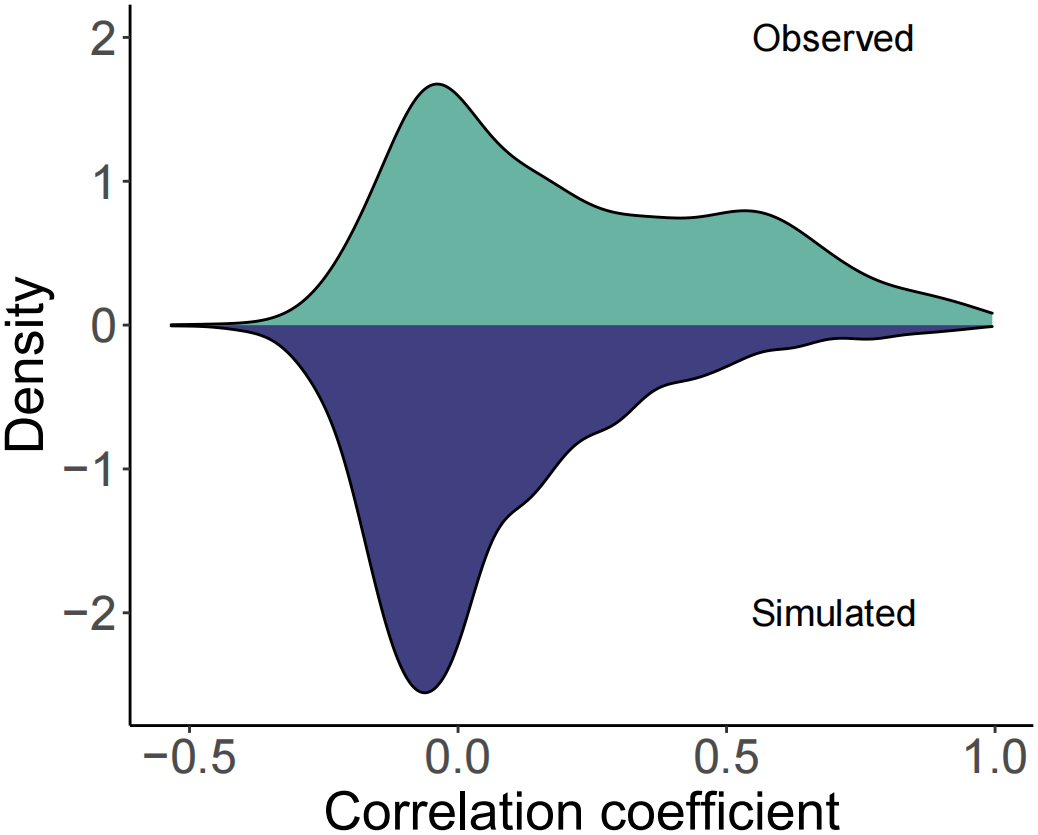


Figure S18. The distribution of expression correlation coefficient between expressed pseudogenes and parent coding genes. The panel above 0 is observed values and below 0 is simulated values. The median observed positive and negative correlation coefficient (R) is 0.33 and -0.08, respectively. The median simulated positive and negative R is 0.17 and -0.09, respectively.


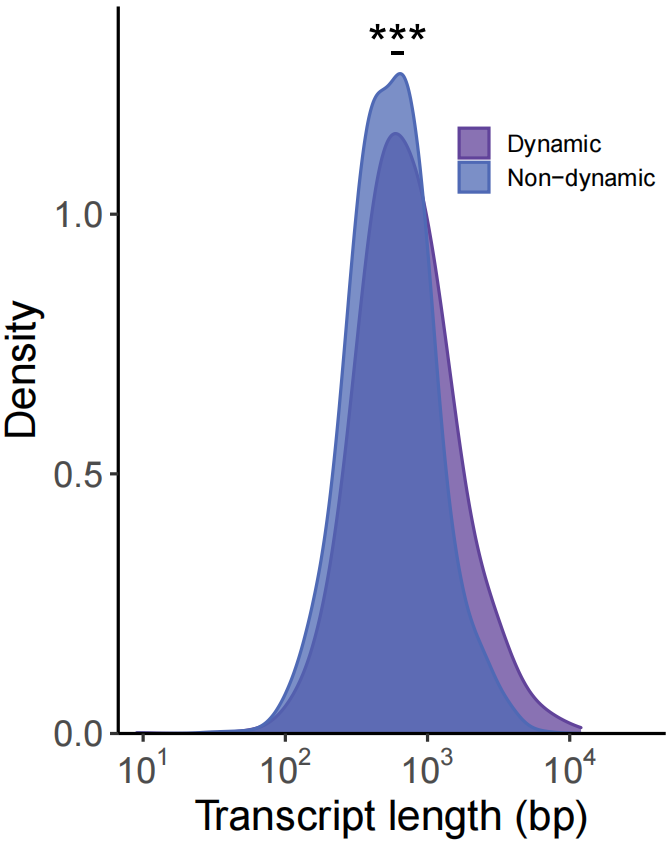


Figure S19. Distribution of transcript length for dynamic and non-dynamic mouse pseudogenes.


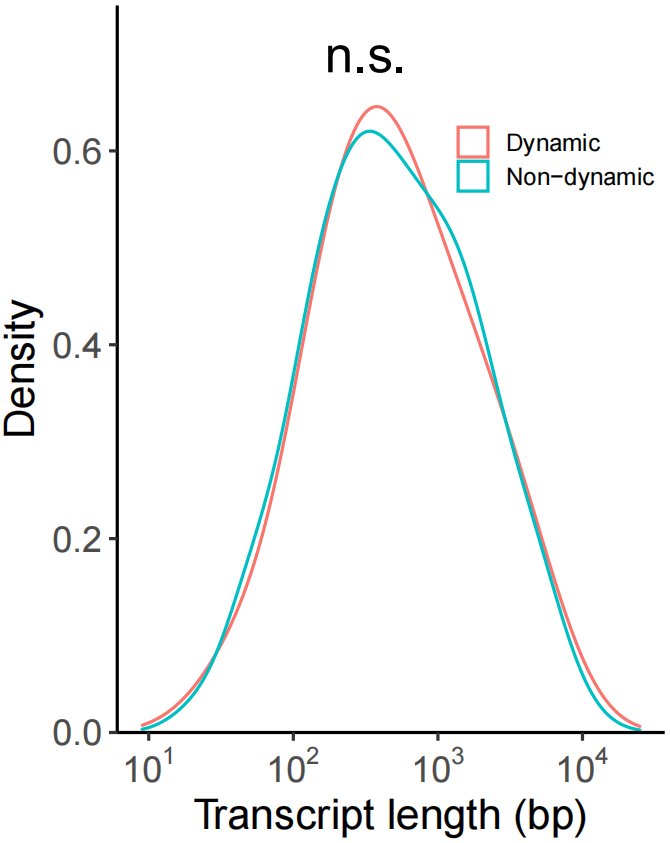


Figure S20. Distribution of transcript length for parent coding genes of dynamic and non-dynamic pseudogenes.


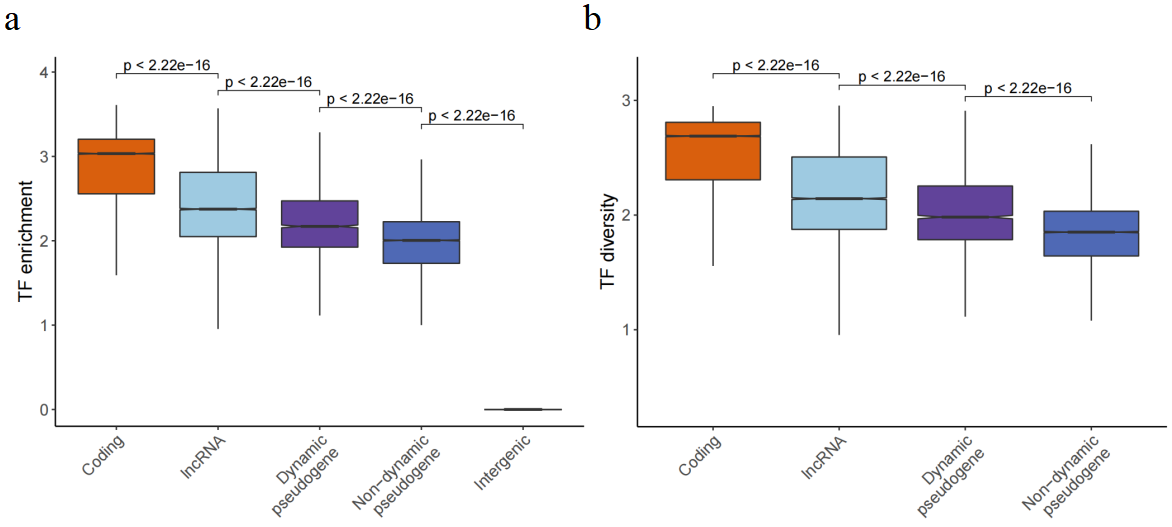


Figure S21. Number and types of TFs overlapping the promoters of protein-coding genes, dynamic pseudogenes, non-dynamic pseudogenes, and randomly shuffled intergenic regions in human genome.


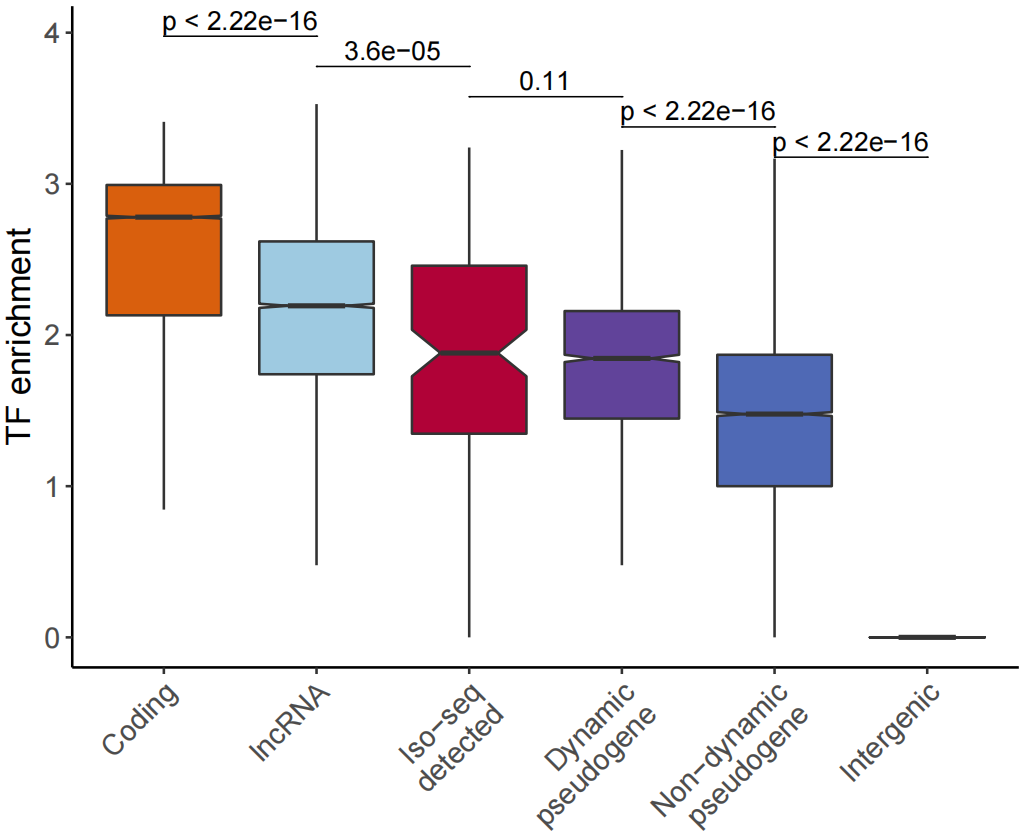


Figure S22. Diversity of TFs overlapping the promoters of protein-coding genes, Iso-seq detected dynamic pseudogenes, dynamic pseudogenes, non-dynamic pseudogenes, and randomly shuffled intergenic regions in mouse genome.


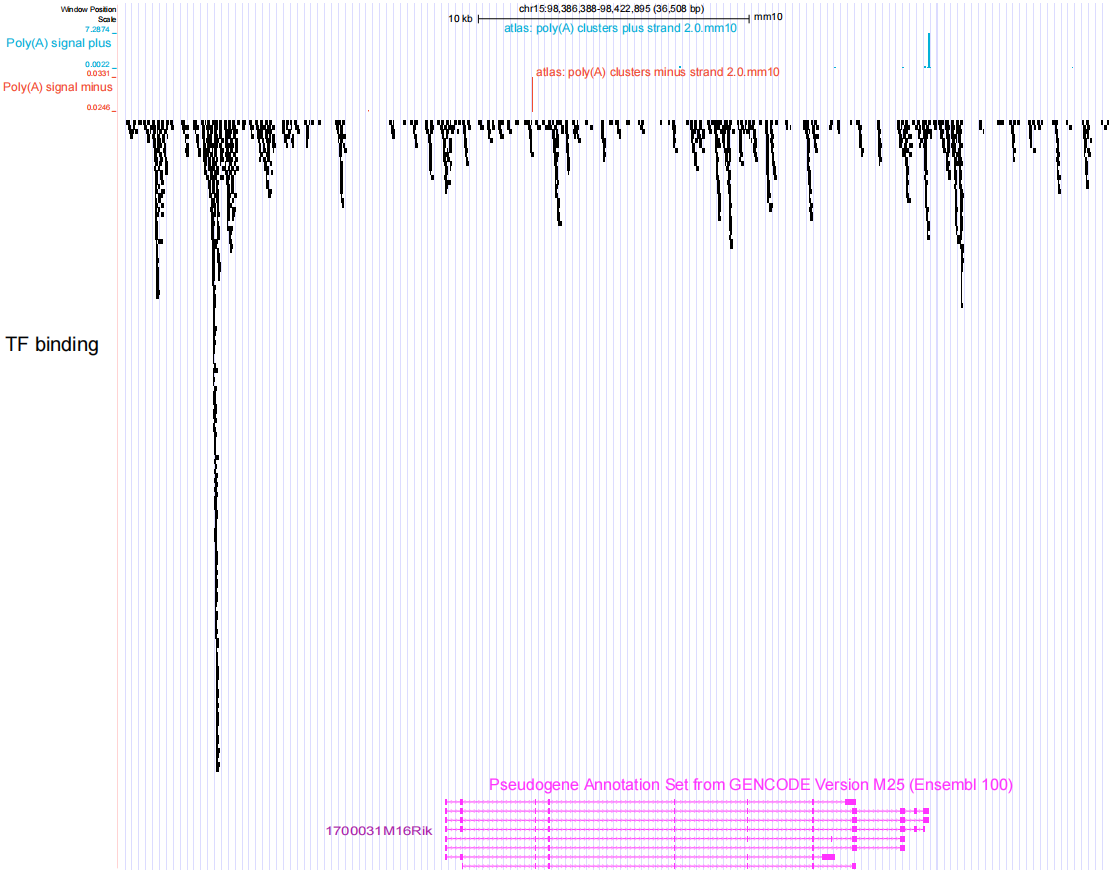


Figure S23. Poly(A) signal and transcription factors binding sites of a genomic locus *1700031M16Rik*. Poly (A) signal on plus and minus strand was shown in blue and red color, respectively. Information about transcription factor (TF) binding sites were integrated from Gene Transcription Regulation Database (see methods).


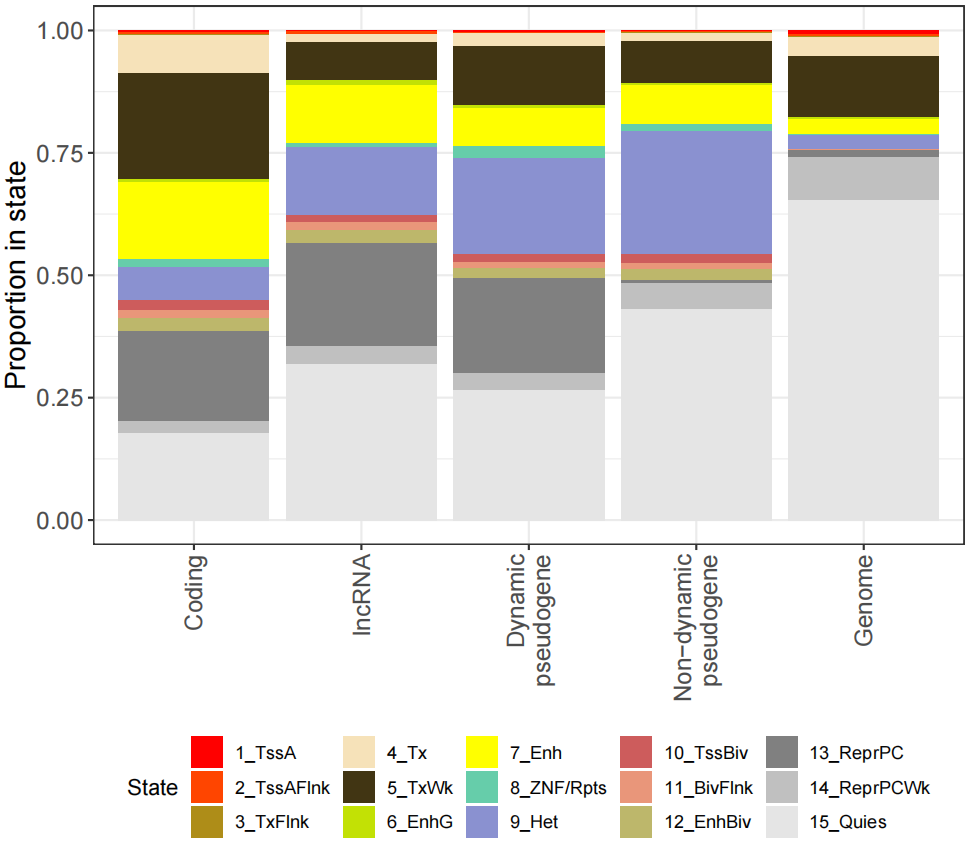


Figure S24. Proportion of state annotated overlapped with promoter of each type of genes annotated with each epigenetic state, summed across all epigenomes.


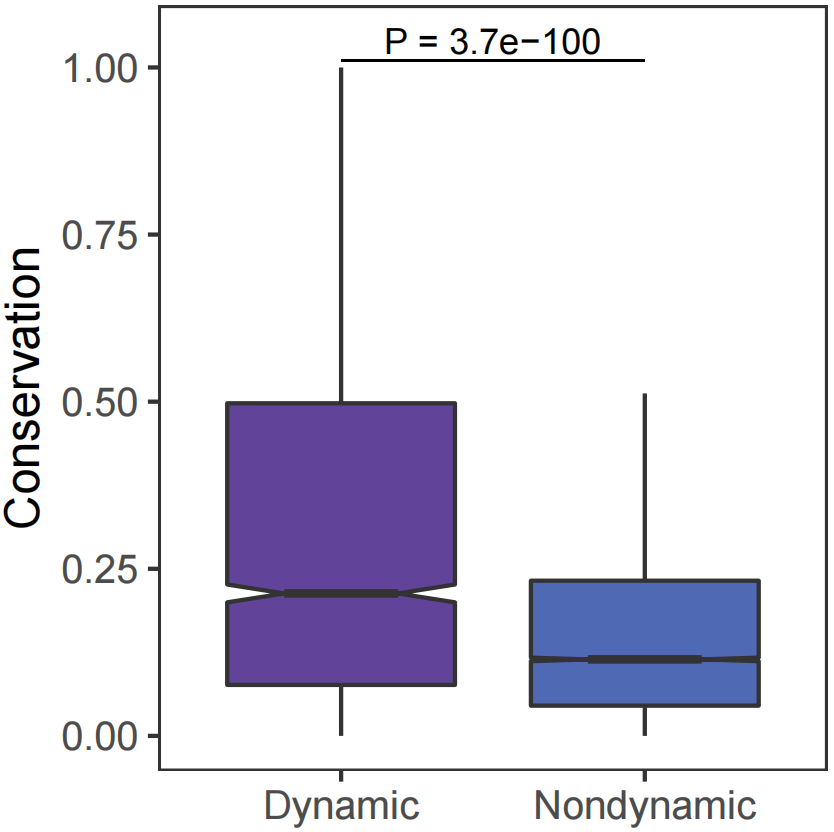


Figure S25. Conservation score of dynamic and nondynamic pseudogenes.


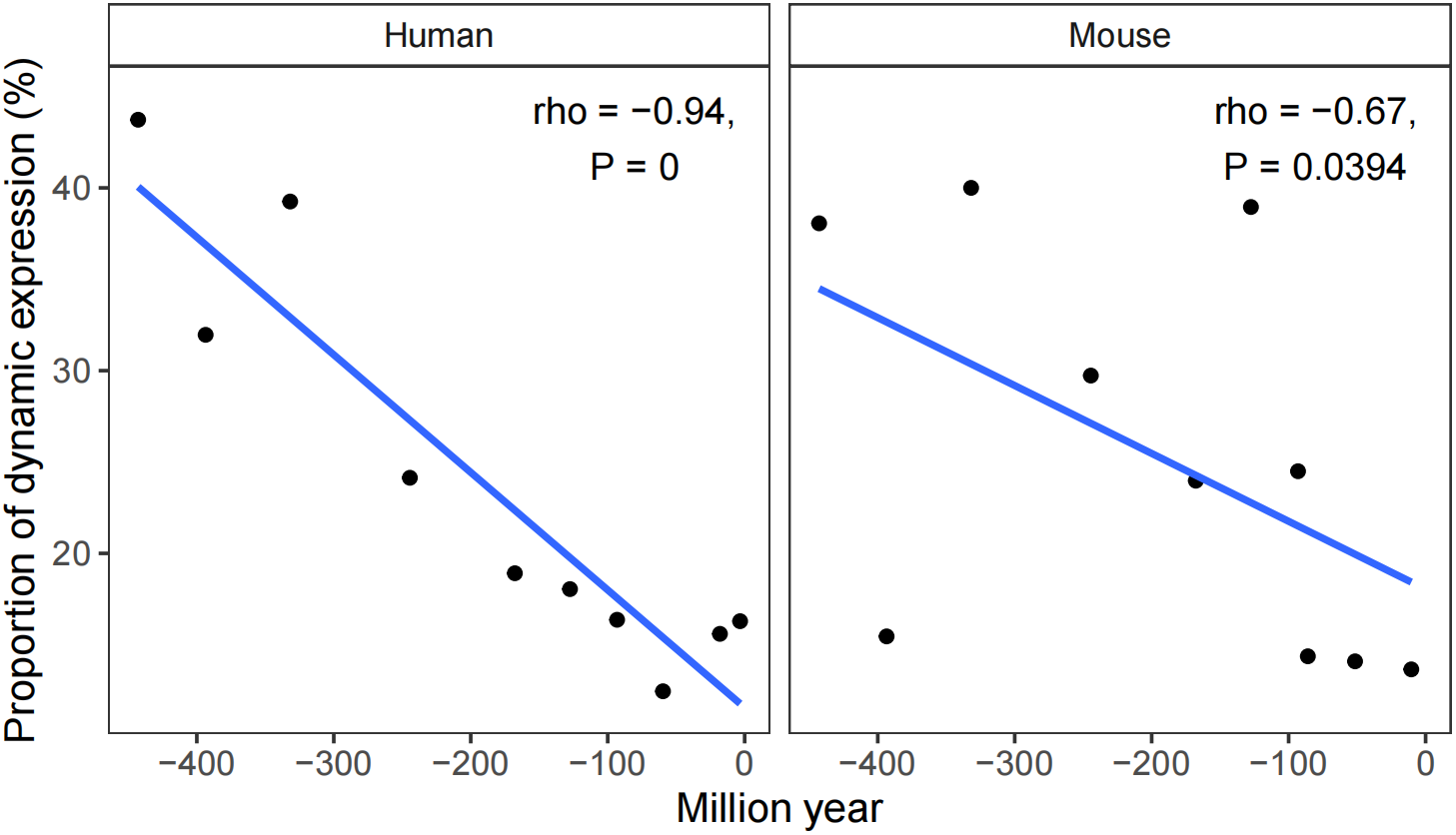


Figure S26. Fraction of dynamic loci for human and mouse pseudogenes of different evolutionary ages.


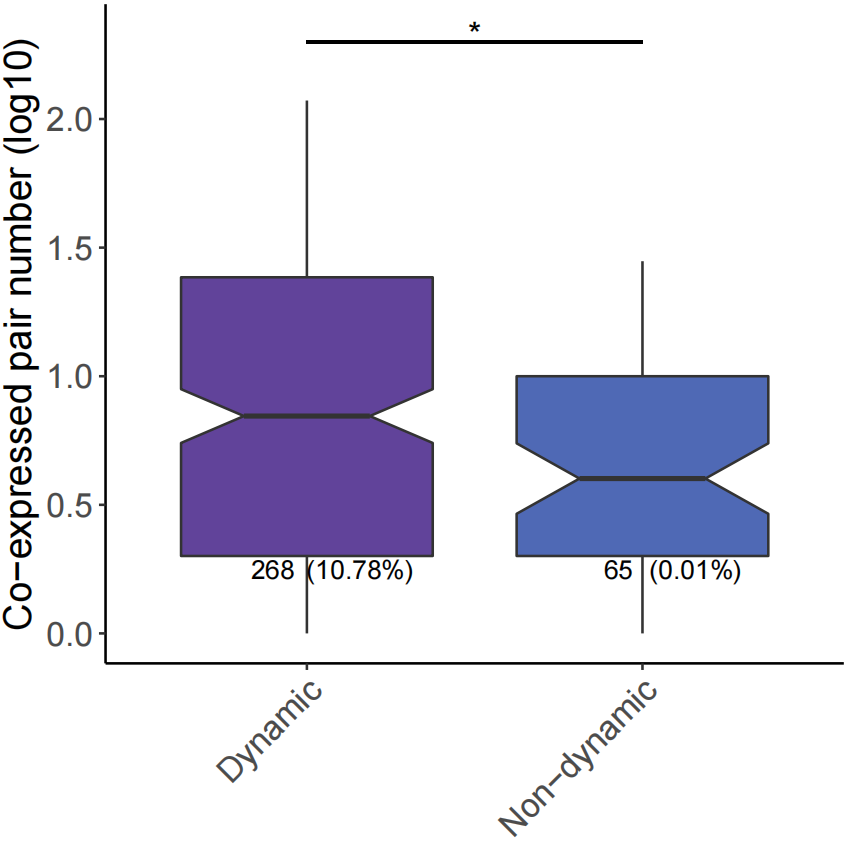


Figure S27. Number of co-expressed pair of dynamic and non-dynamic pseudogenes. The number and percentage in dynamic group represent the number and proportion of dynamic pseudogenes co-expressed with mRNAs, respectively. Likewise, the number and percentage in non-dynamic group represent the number and proportion of non-dynamic pseudogenes co-expressed with mRNAs, respectively.


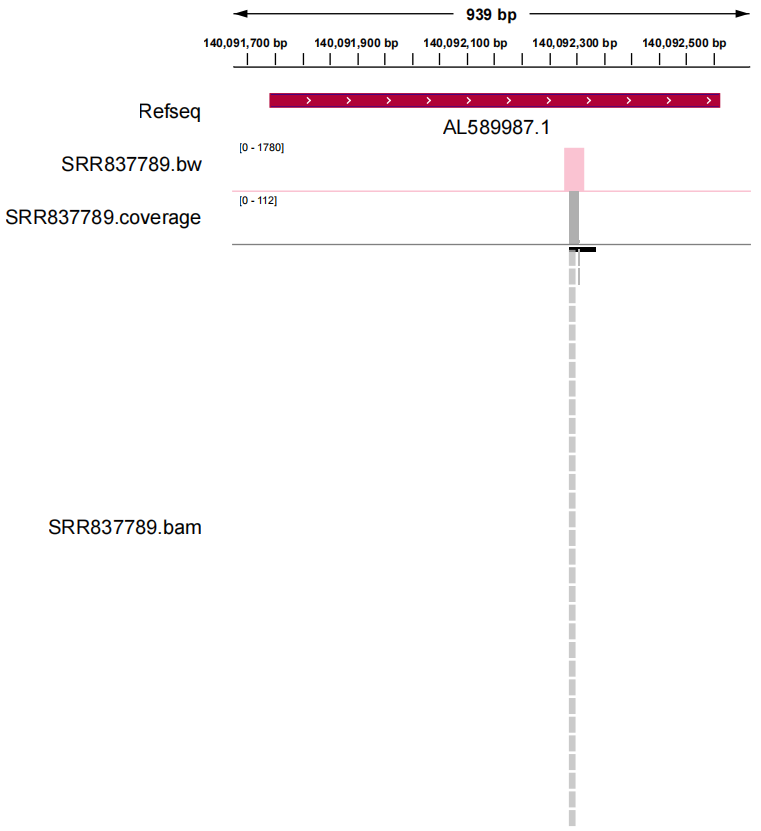


Figure S28. Coverage plot and raw alignments from Ribo-seq reads (SRR837789) for a process pseudogene *AL589987.1* (chrX:140,091,874-140,092,692). A total of 111 unique mapped Ribo-seq reads (MAPQ > 30) were aligned to the locus.


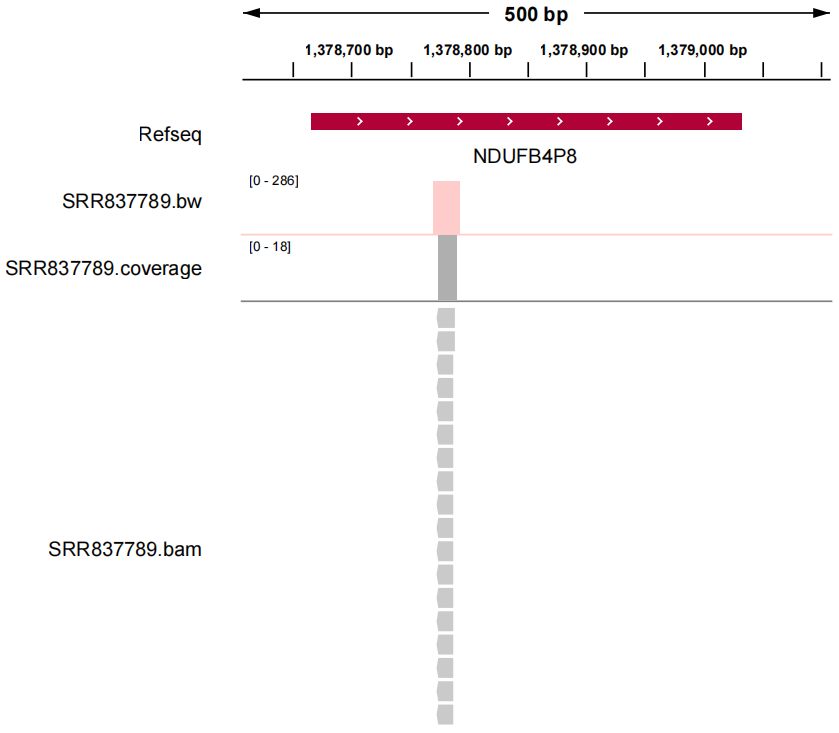
Figure S29. Coverage plot and raw alignments from Ribo-seq reads (SRR837789) for a process pseudogene *NDUFB4P8* (chr1:1,378,666-1,379,032). A total of 18 unique mapped Ribo-seq reads (MAPQ > 30) were aligned to the locus.


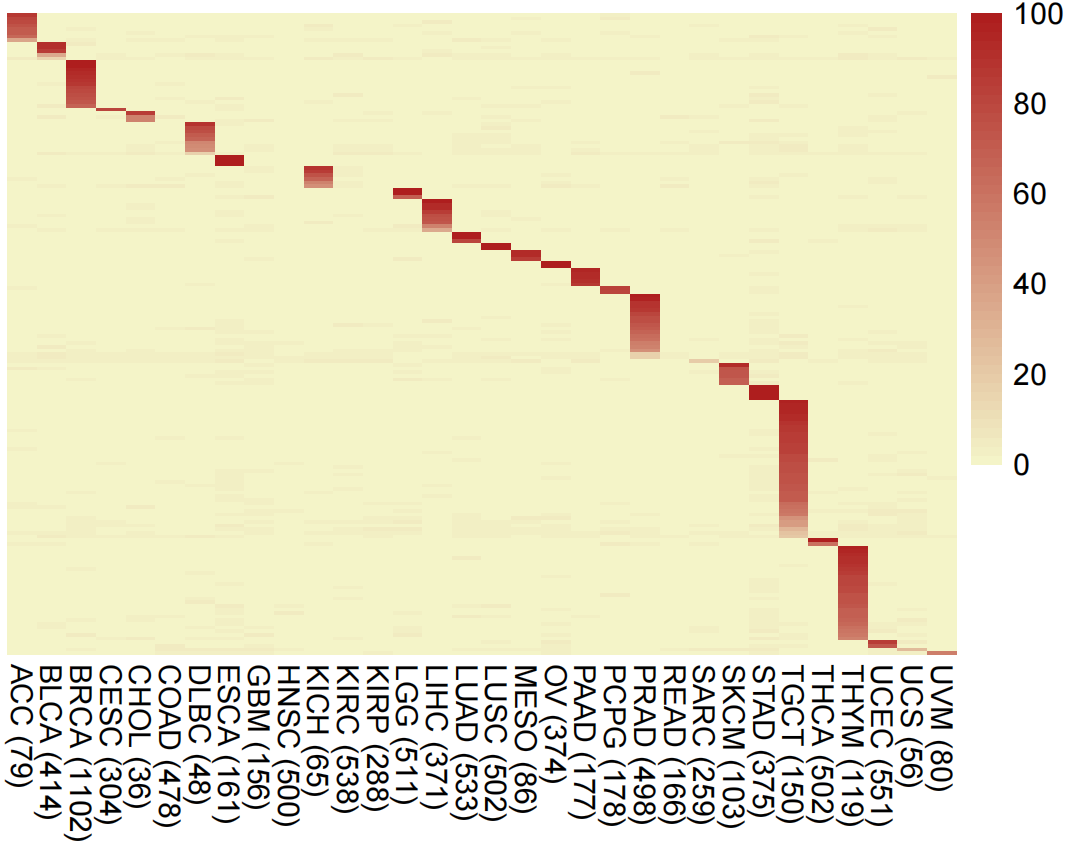


Figure S30. Expression pattern of cancer type-specific pseudogenes across 32 cancer types. The columns represent different cancer types, with the number of samples in bracket.


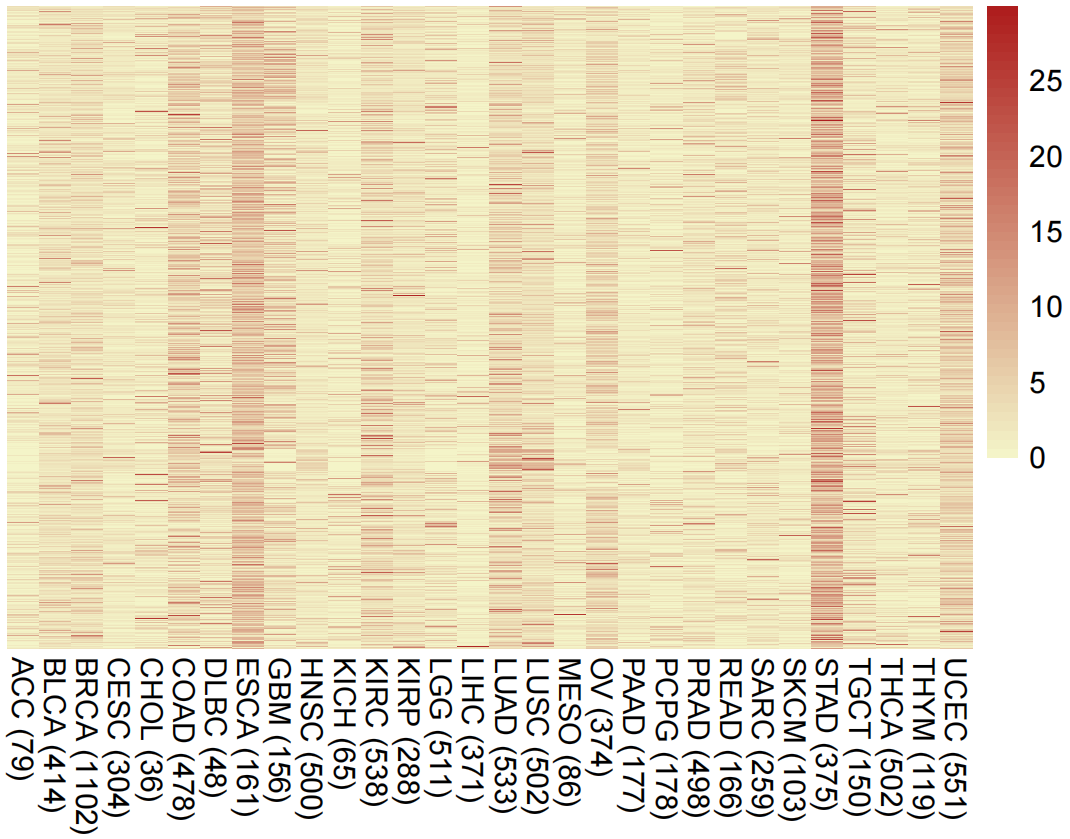


Figure S31. Expression pattern of ubiquitously expressed pseudogenes. The columns represent different cancer types, with the number of samples in bracket.


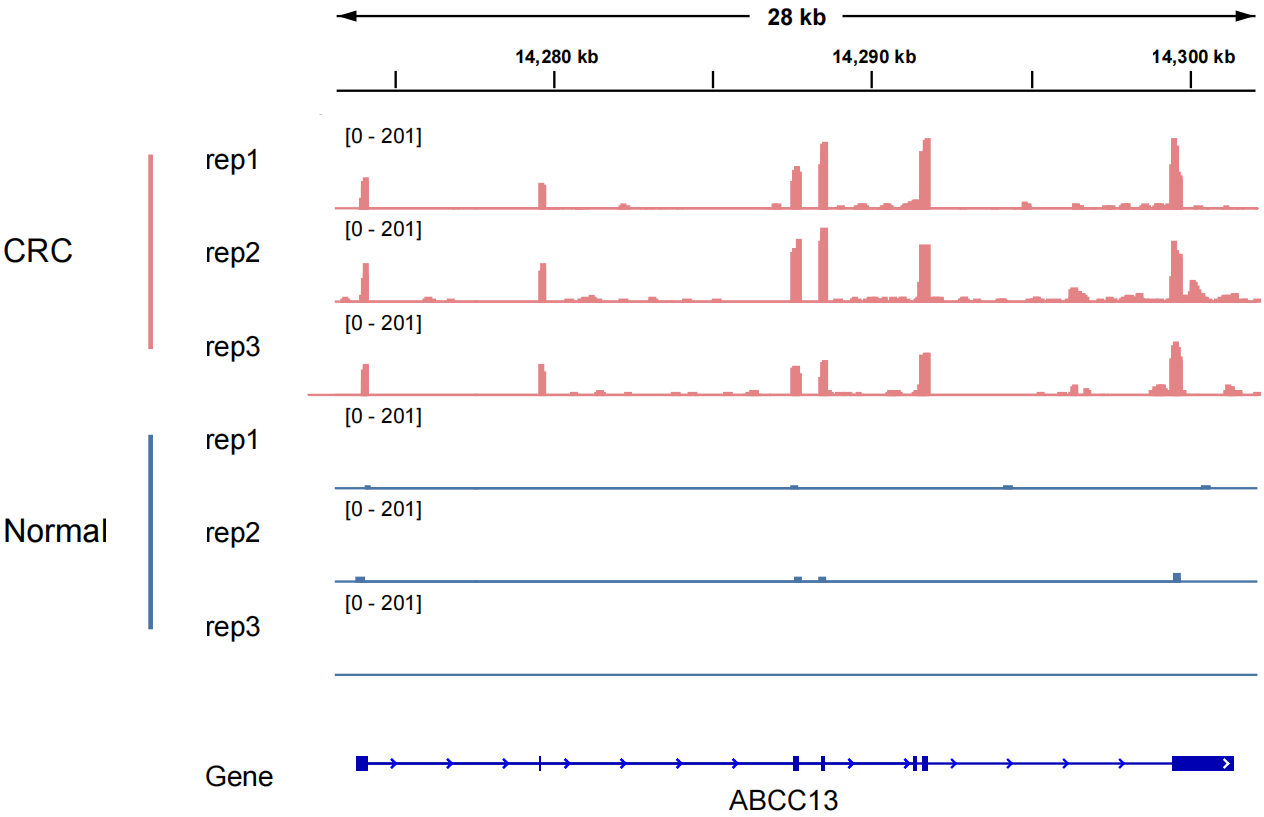


Figure S32. Expression level of *ABCC13* in colorectal cancer (CRC) and adjacent tissues.


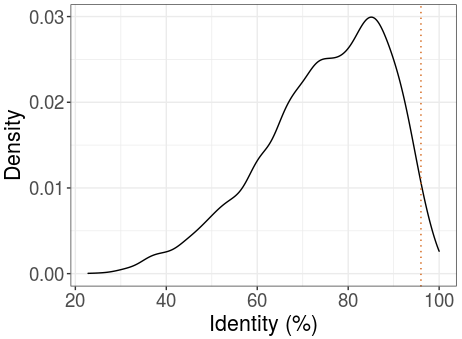


Figure S33. Distribution of sequence identity between pseudogene and parent gene pairs. The red line indicates 96% sequence identity.


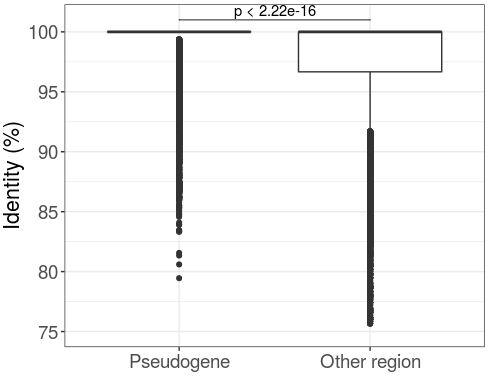


Figure S34. Identity of reads derived from pseudogene loci. The BLAST program was used to calculate the reads identity at pseudogene and other regions.


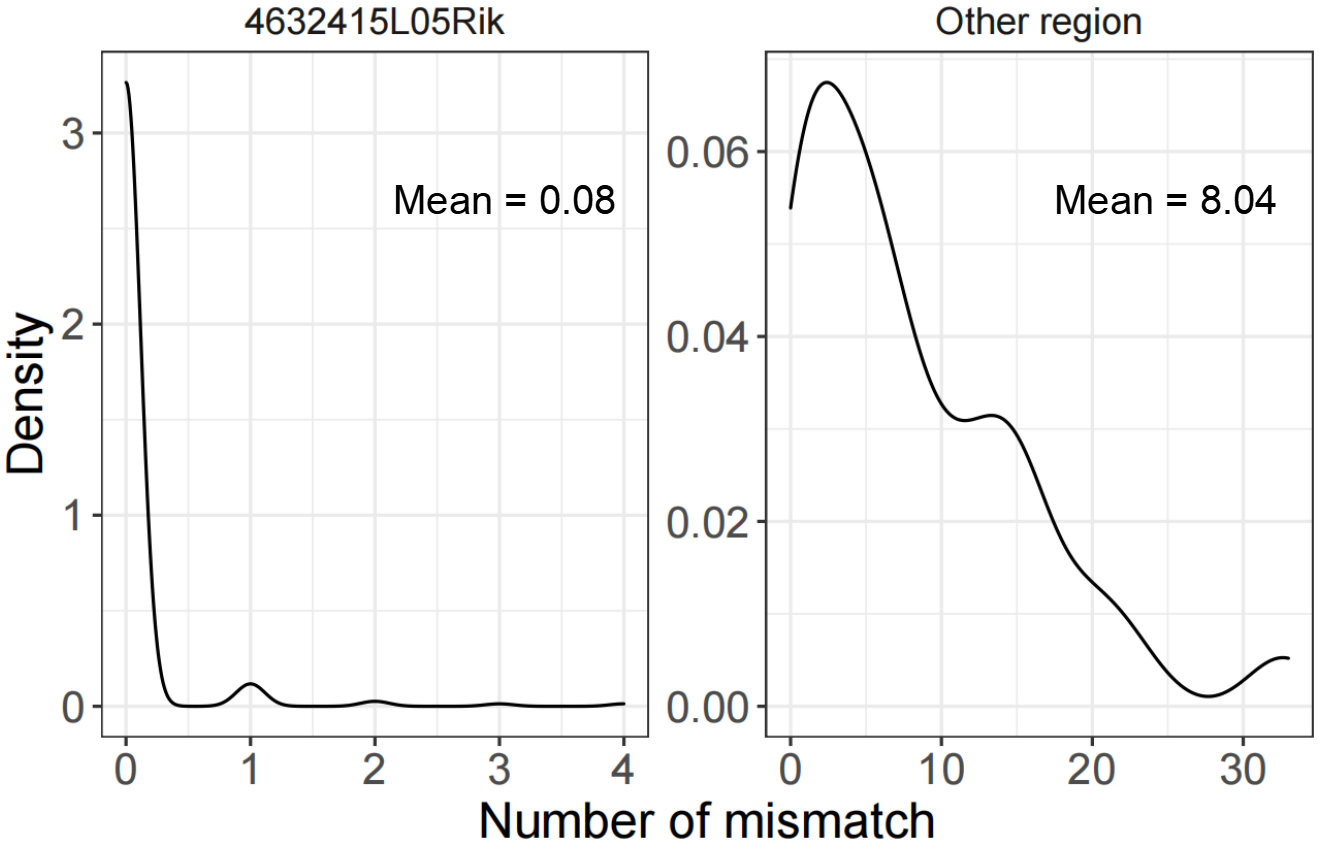


Figure S35. Number of mismatch of reads derived from 4632415L05Rik. The mean number of mismatch of reads at 4632415L05Rik is 0.08 while the mean number of mismatch at other regions (second-best hit) is 8.04.
