# Supplementary material for: Evolution and function of developmentally dynamic pseudogenes in mammals
Source: Genome Biol. 2022 Nov 8;23:235. doi: 10.1186/s13059-022-02802-y (PMC9641868; doi:10.1186/s13059-022-02802-y)
Supplement: Supplementary file 16 — Additional file 16. Review history. [file 13059_2022_2802_MOESM16_ESM.docx]

Review History

**First round of review**

**Reviewer 1**

**Were you able to assess all statistics in the manuscript, including the appropriateness of statistical tests used?**

Yes, and I have assessed the statistics in my report.

**Comments to author:**

Qian et al examine the depth and features of the pseudogene transcriptome using PacBio and Illumina RNA-sequencing. They find a large fraction of pseudogenes may be transcribed in spatially and developmentally regulated patterns. Cancer-specific expression and nearby GWAS SNPs suggest that these pseudogenes may be functionally associated with human development and disease. The study is clear written and presented and could add to the growing body of evidence of pseudogene functionality. The dynamic expression of pseudogenes observed during development is interesting and should in my view be the focus of the paper. However, I have some concerns about the novelty and robustness of the findings presented here.

Specific comments:

-Han et al (PMID: 24999802) examined landscape of pseudogene expression in cancer. Guo et al (PMID: 24699680) and Troskie et al (PMID: 33971925) identified tissue-specific pseudogene transcription. Carelli et al (PMID: 26728716) explored the evolutionary dynamics of retrocopies. The work of the Kaessmann lab and others has extensively covered the enrichment of X-linked pseudogene expression. The advances over these previous studies are not sufficiently clear.

-The PacBio analysis methodology is unclear. How were transcripts identified and compared to ENSEMBL. Was the Iso-Seq pipeline used. I find it very surprising that only 32 pseudogenes were detected as this is far less than comparable studies. What was the depth of sequencing? Read numbers or numbers of flow-cells per sample are not stated. As this data was generated on the Sequel I platform it is likely significantly under-powered to detect pseudogene transcription, which is generally considerably lower than for protein-coding transcripts.

-Despite accurately stating that short-reads are poorly suited to analysing the pseudogene transcriptome apart from one figure all analyses are based on short reads. The PacBio data which should be the main novelty of the study are largely discarded.

-The statement that "Strikingly, approximately 50% of all pseudogenes were expressed in human (49%) and mouse (53%) when we set FPKM > 0.3 as cutoff following a previous study" is a huge overstatement. This should be removed from the abstract and likely paper. This is not a widely used expression level. It is never directly tested in this paper that the read-mappings are completely specific, so it is highly likely that at least some reads originate from the parent gene.

-The dynamic expression of pseudogenes during development is the most novel aspect of this work. Ideally the paper should be refocused to emphasise these results.

-It is unsurprising that pseudogenes have higher scores from in silico coding potential calculators. Each pseudogene is derived from an ORF and as such many retain large ORFs even if not translated.

-It is unclear if the Ribo-seq analysis is restricted to pseudogenes that display the characteristic 3-nucleotide signature, indicating that the transcript is actively translated (rather than just co-purified with the ribosome). It is also unclear as this is short-read data that this analysis is specific to the pseudogenes and does not suffer from the mismapping of short-reads. At a minimum raw alignments should be shown indicating specificity of this analysis.

-More example loci should be shown throughout the paper. It is assumed throughout that the mappings of secondary datasets are specific despite the notorious difficulty of analysing pseudogene transcripts with short reads. Adequate demonstration of the specificity of this using genome browser images i.e Ribo-seq, developmental timecourse, cancer-specificity.

-The GEO repository currently does not seem to include the raw sequencing data. This data is essential to evaluate the veracity of the findings.

-Scripts used to generate these results must be be publicly available under a github repository not available on reasonable request.

**Reviewer 2**

**Were you able to assess all statistics in the manuscript, including the appropriateness of statistical tests used?**

Yes, and I have assessed the statistics in my report.

**Comments to author:**

In this article the authors have presented systematically examines pseudogenes in human and mouse, identifying their evolution across vertebrates and hypothesise that 50% of pseudogenes could be transcribed and predict 19% of pseudogenes could be translated . Developmentally dynamic pseudogenes are found to be conserved and involved in complex expression.

This is an extremely thorough analysis of human and mouse pseudogenes, however some of the narrative is a little confusing and therefore how the authors arrive precisely at the 50% figure of expressed pseudogenes is a little confusing. My review comments are split in major and minor points and given below and I have tried to highlight where more clarity is needed in the analysis :

Major points:

1. In the results pg5 line 97 the author mention that the pseudogenes are assigned by ensemble compara . However these are obtained from the GENCODE project https://www.gencodegenes.org/human/stats.html and manually curated and this should be mentioned as the biotypes (unitary, processed,unprocessed etc) are obtained from this annotation process

2. The longer isoseq transcript that overlapped the processed pseudogenes , were any of these transcripts spliced ?

3. The annotation of the 3110053b16RIK (fig2C) appears a little confusing as it contains both pseudogene and zinc finger that overlaps the pseudogene and may be the reason for the detection of expression in all tissues. Would it be possible to use another example see: https://www.ensembl.org/Mus_musculus/Gene/Summary?db=core;g=ENSMUSG00000085642;r=12:20924126-20971263

4. The FPKM level appears very low and could be the reason that the level of expressed pseudogenes is 5 times higher than ENSEMBL that used 2 FPKM as using small reads with a mismatch error rate can cause misalignment. It would be useful to perform a range of cutoffs from 0.3 FPKM, 0.6 FPKM and 1 FPKM and 2 FPKM and look at the distribution of detected pseudogenes . It would be interesting if the isoseq confirmed pseudogenes can be identified as transcribed using the higher 1 or 2 FPKM threshold and using only the short read transcriptomic data as a control . The publication cited for the use of FPKM 0.3 actually in the method it states "the pseudogenes with detectable expression were defined as those with an average RPKM ≥ 1 across all samples in each cancer type, as used in the literature" so is more stringent cutoff than has been used for this analysis and I would recommend the authors use a more stringent cutoff

5. It would be useful to reproduce figure S8 with different RPKM cutoffs to see whether the distribution of expressed pseudogenes changes across biotypes when a more stringent FPKM is used as there are only 243 unitary pseudogene and yet it appears that nearly over 120 of these are transcribed and can be detected yet only 1 unitary pseudogene was detected using isoseq

6. The comment on Pg 10 line 213 " the highest pseudogene expression was in testis" maybe should be clarified as do the authors not attribute this to leaky expression and potentially not biologically relevant since "the massive chromatin remodeling during spermatogenesis causes leaky expression" (Soumillon et al. 2013 especially since a low FPKM was used for detection.

7. The graph S17 that indicates that dynamic developing pseudogenes are longer than non DDPs looks not very convincing S18-19 as the majority overlap in size, Were any of the isoseq sequences identified as DDPs and can the authors show the actual increase in TFs /extension or preserved polyA sites/signals? It would also be useful to see which biotype category the DDPs are subcategorized into, eg are they mostly processed pseudogenes?

8. Brosch et al identified 9 processed pseudogenes mouse using mass spec data from peptide atlas that is publicly available, so why could the authors not use this data to screen their pseudogenes ? Also in the 19% pseudogenes expressed do the 9 processed pseudogenes from Brosch et al appear ?

9. What would help the readers navigate all the complex work that the authors have done would be a summary table of the number of mouse and mouse pseudogenes analysed by the different analyses (RNAseq, isoseq, Riboseq, CPAT) and actual number of pseudogenes detected (subdivided by biotypes) as it would be interesting to identify if the majority of DDPs were unprocessed pseudogenes and what percentage of processed pseudogenes were translated etc.

Minor points :

1. Pg4 line 72 processed Adh gene (jingwei), is there a reference missing here ?

2. Pg4 line 78 please expand what is refereed to concerning ceRNA behaviour.

3. page 7 line 142 why the publication by Troskie et al 2021 support issue of short reads hindering identifying transcribed pseudogenes as is about long reads

4. Pg 20 line 433 typo Pipeline

**Authors Response**

**Point-by-point responses to the reviewers’ comments:**

Reviewer #1:

Comment: Qian et al examine the depth and features of the pseudogene transcriptome using PacBio and Illumina RNA-sequencing. They find a large fraction of pseudogenes may be transcribed in spatially and developmentally regulated patterns. Cancer-specific expression and nearby GWAS SNPs suggest that these pseudogenes may be functionally associated with human development and disease. The study is clear written and presented and could add to the growing body of evidence of pseudogene functionality.

*Response: We appreciate your positive remarks on the manuscript.*

Comment: The dynamic expression of pseudogenes observed during development is interesting and should in my view be the focus of the paper.

*Response: Yes, the dynamic expression of pseudogenes is our focus.*

Comment: However, I have some concerns about the novelty and robustness of the findings presented here.

*Response: We sincerely appreciate your constructive suggestions, which we address in details as below.*

Specific comments:

Comment: -Han et al (PMID: 24999802) examined landscape of pseudogene expression in cancer. Guo et al (PMID: 24699680) and Troskie et al (PMID: 33971925) identified tissue-specific pseudogene transcription. Carelli et al (PMID: 26728716) explored the evolutionary dynamics of retrocopies. The work of the Kaessmann lab and others has extensively covered the enrichment of X-linked pseudogene expression. The advances over these previous studies are not sufficiently clear.

*Response: Thank you very much for bringing these important studies on pseudogenes to our attention.*

*The identification and characterization of developmentally dynamic pseudogenes (DDPs), as well as the integration of PacBio full-length sequencing and Illumina time-course sequencing data, are our advances. Cardoso-Moreira et al (PMID: 31243369) indicated that evolutionary analysis should be performed within a developmental framework since evolutionary and developmental processes are intertwined. To systematically characterizing the function and evolution of mammalian pseudogenes in a single framework, we integrated PacBio full-length sequencing and Illumina time-course sequencing data and revealed that the expressed pseudogenes contributed to organ identity and mirror developmental trajectory, as observed for protein-coding genes and lncRNAs. Moreover, we nominated the DDPs with signatures of functional enrichment and subsequently found that they might represent an additional regulatory layer. To clarify our novelty, we introduced these publications and summarized above points in the Background on pages 4-5 (lines from 74 to 100) as below.*

*“With the availability of throughput sequencing recently, a growing body of evidence has uncovered the functions of some pseudogenes under physiological and pathological conditions [13-18]. For example, some studies have shown that pseudogenes played important roles in cancer progression and could stratify the subtype of kidney cancer [19, 20]. Furthermore, they were reported to exhibit tissue-specific expression, suggesting the distinct regulatory programs [21, 22]. Nevertheless, the studies of pseudogene transcription, the first step to generate functional RNA, are precluded by the limited capacity of short-read sequencing. A recent work applied long-read PacBio sequencing to identify functional human pseudogenes and provided the evidence that pseudogenes regulated the cellular transcriptome [23]. Although above findings significantly advanced our knowledge about pseudogene functions, the majority of these studies focus on the function of pseudogenes in disease and cancer contexts and are limited to the interplay between pseudogenes and parent coding genes, such as competing endogenous RNAs (ceRNA) behavior, a phenomenon that an RNA molecular could regulate other RNAs by competing for identical microRNAs [18, 24]. Alternatively, only a subset of tissue expression data was covered in their analysis, which might unavoidably underrate pseudogene expression abundance due to the spatiotemporal gene expression pattern [25, 26]. Meanwhile, there has been limited systematic functional characterization of mammalian pseudogenes and in particular, the contribution of pseudogene to organ development is largely unknown. Considering this, in this study, we systematically inferred the origin time of human and mouse pseudogenes and characterized their evolution pattern. Using PacBio full-length sequencing data, we refined the gene structures and identified full-length pseudogene transcripts. In combination with deep Illumina sequencing data and public developmental RNA-seq data [25], we dramatically expanded the analyzed data set and profiled genome-wide pseudogene expression patterns. Additionally, we prioritized developmentally dynamic pseudogenes with signatures of functional enrichment, found that they might represent an additional regulatory layer, and determined their implications in disease and cancer biology.”*

Comment: -The PacBio analysis methodology is unclear. How were transcripts identified and compared to ENSEMBL. Was the Iso-Seq pipeline used.

*Response: We have extended the details of PacBio analysis in the Method section (page 26, lines 555-567) as below.*

*“PacBio data processing*

*The Raw data were subjected to SmrtLink Pipeline ccs (v5.0.0) for self-correction to obtain full-length circular consensus sequencing (CCS) reads based on PacBio recommended pipeline. We applied the SmrtLink Pipeline Cluster subprogram to cluster the full-length non-chimeric sequences into full-length transcripts (longer than 300bp) and retained 899,237 full-length transcripts supported by at least one full-length non-chimeric sequence through Isoseq 3 [78]. The LoRDEC program (v0.7) [79] was leveraged to correct the full-length transcripts based on the results of Illumina RNA-seq to improve the accuracy of the third generation transcripts. After being corrected by LoRDEC using next-generation RNA-seq data, the third generation transcripts were mapped to reference genome (Ensembl 98) through minimap2 (version, 2.17-r954-dirty) [80]. Then the redundant transcripts model (GFF3 format) for each high-quality non-chimeric CCS read from minimap2 output were collapsed using the Python script (collapse_isoforms_by_sam.py) from cDNA_cupcake ((https://github.com/Magdoll/cDNA_Cupcake) to generate a non-redundant set of transcript model (termed as Iso set). Gffcompare (GFF Utilities, v0.11.2) [81] was used to compare each isoform in Iso set with existing mouse Ensembl gene models (termed as reference set). The genes annotated in the reference set but not overlapped with Iso-seq annotation at the same strand were merged with Iso-seq annotation to form the final annotation. The pseudogene transcripts were identified and classified as those assigned the name of reference pseudogenes by Gffcompare.”*

Comment: I find it very surprising that only 32 pseudogenes were detected as this is far less than comparable studies.

*Response: Thanks for your concern. We have checked our Iso-seq pipeline and detected a total of 177 pseudogenes, which is comparable with previous studies (Troskie et al, PMID: 33971925). All the related results have been updated and checked to ensure the consistency and accuracy. We have also uploaded the Iso-seq annotation model to our GEO submission (https://ftp.ncbi.nlm.nih.gov/geo/series/GSE176nnn/GSE176018/suppl/GSE176018_mouse_final.gtf.gz).*

Comment: What was the depth of sequencing? Read numbers or numbers of flow-cells per sample are not stated. As this data was generated on the Sequel I platform it is likely significantly under-powered to detect pseudogene transcription, which is generally considerably lower than for protein-coding transcripts.

*Response: We sequenced high quality RNA from somatic and gonad tissues of two sexes of the mouse on the Sequel I platform, including 4 RNA libraries (6 Pacbio flow-cells) and 94.4 Gb, and produced 1,946,228 raw reads. Among the 49,914 full length transcripts, 332 were derived from pseudogene loci. We have put the results into the manuscript (page 25, lines 545-546, page 26, line 563).*

Comment: -Despite accurately stating that short-reads are poorly suited to analysing the pseudogene transcriptome apart from one figure all analyses are based on short reads. The PacBio data which should be the main novelty of the study are largely discarded.

*Response: We agree with the reviewer on the significance of PacBio data. Following your suggestion, we have added more results generated from PacBio data, including investigating the abundance of PacBio detected pseudogenes subdivided by biotype, analyzing the overlap degree between PacBio detected pseudogenes and transcribed pseudogenes annotated by reference, showing more loci that were detected by PacBio and their expression pattern, comparing the expression level between PacBio detected pseudogenes and others, examing the regulatory complexity between DDPs and PacBio detected DDPs. We visualized these results in the updated figure 2c-f, figure 5b and g, figure S2, and S20, and added the description on page 8 (lines 159-173).*

Comment: -The statement that "Strikingly, approximately 50% of all pseudogenes were expressed in human (49%) and mouse (53%) when we set FPKM > 0.3 as cutoff following a previous study" is a huge overstatement. This should be removed from the abstract and likely paper. This is not a widely used expression level.

*Response: We have removed this sentence and analogous statement throughout the manuscript. Additionally, we have added a range of FPKM cutoffs to check the distribution and sensitivity of detected pseudogenes under different cutoffs (shown in figure 2g) and used a more stringent cutoff (FPKM ≥ 1) for expression analysis.*

Comment: It is never directly tested in this paper that the read-mappings are completely specific, so it is highly likely that at least some reads originate from the parent gene.

*Response: Thank you. We only considered the unique mapped reads (MAPQ > 30) for subsequent analysis.*

Comment: -The dynamic expression of pseudogenes during development is the most novel aspect of this work. Ideally the paper should be refocused to emphasise these results.

*Response: We thank the reviewer for highlighting the importance of the dynamic expression of pseudogenes during development. To emphasize these results, we highlighted that the contribution of pseudogenes to development is largely uncertain in the abstract section (page 2, line 27), changed the term into “Developmentally dynamic expression” in keywords, stressed DDPs with signatures of enrichment for functionality in the background (page 5, lines 97-99, 104), and changed the last two subtitles in the Result section to “DDPs are more likely to be translated” and “DDPs conduce to cancer transcriptome alterations”, respectively. Thanks to your suggestions, we feel the logic of our manuscript is much clearer, flowing from evolutionary analysis, subsequent expression investigation, to systematic characterization of DDPs (contribution of DDPs to organ development, regulation, translation, and cancer biology). Moreover, to clarify the focus of DDPs to the reader, we emphasized the functional importance of DDPs in the conclusion section and pointed out that these DDPs could be prior candidates to investigate pseudogene biology on pages 22-23 (lines 481-489) as below.*

*“Here, we analyzed the evolution and expression of mammalian pseudogenes within a developmental framework and provided a comprehensive expression profile of pseudogene transcriptomes by integrating PacBio long-read sequencing and large-scale RNA-seq data. We also identified the DDPs with enrichment of functional features, provided proof-of-principle evidence that DDPs contributed to organ development and might represent a new regulatory layer, and associated these DDPs with putative functions (i.e., metabolic process and DNA modification). Future studies combining experiments and emerging sequencing technologies will further uncover the regulatory profiles of these DDPs and elucidate their phenotypic consequences and underlying molecular mechanisms.”*

Comment: -It is unsurprising that pseudogenes have higher scores from in silico coding potential calculators. Each pseudogene is derived from an ORF and as such many retain large ORFs even if not translated.

*Response: Thank you very much for your comments. To highlight this point, we have updated the text on page 17 (lines 364-367) as below.*

*“Meanwhile, we found that 3,645 (24%) pseudogenes were identified as potential translated candidates. The higher translation potential of pseudogenes than lncRNAs (Fisher exact test, P < 2.2×10-16) could be retained from their parent protein coding genes. ”*

Comment: -It is unclear if the Ribo-seq analysis is restricted to pseudogenes that display the characteristic 3-nucleotide signature, indicating that the transcript is actively translated (rather than just co-purified with the ribosome). It is also unclear as this is short-read data that this analysis is specific to the pseudogenes and does not suffer from the mismapping of short-reads. At a minimum raw alignments should be shown indicating specificity of this analysis.

*Response: Thanks for raising the important question about the specificity of ribosome protected fragments. The fragments were about 30 nt and Ji et al (PMID: 26687005) has demonstrated that it is rare for nucleotide identicalness between pseudogenes and parent coding genes to occur over this length. Additionally, Moqtaderi et al (PMID: 20418883) have handled the same issue where there was much higher similarity between related genes (different tRNA genes). Moreover, in our analysis, only uniquely mapped reads were retained and used for subsequent quantification. Following the reviewer’s suggestion, we displayed examples in figure S26-27, two processed pseudogenes AL589987.1 and NDUFB4P8, to which 111 and 18 unique mapped reads (MAPQ > 30) were aligned, respectively. To further filter indeed translated ones, we collected 24,724,526 transcripts with actively translated open reading frames (ORFs) detected by RibORF, which uses Ribo-seq data and combines alignment of ribosomal A-sites, characteristic 3-nt periodicity, and uniformity across codons. The pseudogenes with FPKM higher than 1 in ribo-seq data and harbored at least one ORF were considered to be translated. 2,286 out of 2,941 pseudogenes with FPKM ≥ 1 contained at least one active ORF. We have updated the results and summarized the detailed ORF information in Supplementary Table 6.*

Comment: -More example loci should be shown throughout the paper. It is assumed throughout that the mappings of secondary datasets are specific despite the notorious difficulty of analysing pseudogene transcripts with short reads. Adequate demonstration of the specificity of this using genome browser images i.e Ribo-seq, developmental timecourse, cancer-specificity.

*Response: Thanks for your helpful suggestions. We have added the examples of tissue-specific pseudogenes (Fig. 2e and Fig. S2), diverse expression patterns of DDPs during development (Fig. 4b), enrichment of TF binding sites at promoter of DDP (Fig. S21), translated DDP (Fig. S26-27), and cancer-specificity (Fig. S30) using genome browser images.*

Comment: -The GEO repository currently does not seem to include the raw sequencing data. This data is essential to evaluate the veracity of the findings.

*Response: We have submitted the data to GEO (https://www.ncbi.nlm.nih.gov/geo/query/acc.cgi?acc=GSE176018), which has submitted the raw sequencing data to SRA (https://trace.ncbi.nlm.nih.gov/Traces/sra/?study=SRP322355). All the data are publicly available now.*

Comment: -Scripts used to generate these results must be be publicly available under a github repository not available on reasonable request.

*Response: All scripts used in this manuscript have been deposited to github (https://github.com/Shengqian95/DynamicPseudogene). Thanks again for your efforts and helpful suggestions.*

Reviewer #2:

Comment: In this article the authors have presented systematically examines pseudogenes in human and mouse, identifying their evolution across vertebrates and hypothesise that 50% of pseudogenes could be transcribed and predict 19% of pseudogenes could be translated . Developmentally dynamic pseudogenes are found to be conserved and involved in complex expression.

This is an extremely thorough analysis of human and mouse pseudogenes,

*Response: Thanks for your positive feedback.*

Comment: however some of the narrative is a little confusing and therefore how the authors arrive precisely at the 50% figure of expressed pseudogenes is a little confusing. My review comments are split in major and minor points and given below and I have tried to highlight where more clarity is needed in the analysis :

*Response: Thanks for your constructive comments. Please see our response in details as below.*

Major points:

Comment: 1. In the results pg5 line 97 the author mention that the pseudogenes are assigned by ensemble compara. However these are obtained from the GENCODE project https://www.gencodegenes.org/human/stats.html and manually curated and this should be mentioned as the biotypes (unitary, processed,unprocessed etc) are obtained from this annotation process.

*Response: Thanks for your kind notice. We have changed the Ensemble compara to GENCODE project on page 6 (line 109) and also highlighted this point in methods on page 23 (lines 493–495) as below.*

*“The human and mouse pseudogene annotations, including their coordinates and biotypes (processed, unprocessed, unitary, and polymorphic pseudogene) generated and manually curated by GENCODE project [27], were downloaded from ENSEMBL compara [35]. ”*

Comment: 2. The longer isoseq transcript that overlapped the processed pseudogenes , were any of these transcripts spliced ?

*Response: Thanks for your comment. Here, we detected two processed pseudogenes with spliced transcripts and showed an example of a processed pseudogene, 2810025M15Rik, in Figure R1.*

Comment: 3. The annotation of the 3110053b16RIK (fig2C) appears a little confusing as it contains both pseudogene and zinc finger that overlaps the pseudogene and may be the reason for the detection of expression in all tissues. Would it be possible to use another example see: https://www.ensembl.org/Mus_musculus/Gene/Summary?db=core;g=ENSMUSG00000085642;r=12:20924126-20971263

*Response: Thank you very much for your careful check. We have replaced this pseudogene with another three examples, including 4933401B06Rik, Gm13857, and 4632415L05Rik, which were detected by Iso-seq and did not overlap with any other gene structures as below (page 8, lines 163-167).*

*“Illustrative examples were the transcribed pseudogenes 4933401B06Rik, Gm13857, and 4632415L05Rik, which do not overlap with any other gene structures. The first two were exclusively expressed in testis, while the third one (4632415L05Rik) exhibited a higher expression level in brain and ovary than in other tissues (Fig. 2e, Additional file 1: Figure S2).”*

Comment: 4. The FPKM level appears very low and could be the reason that the level of expressed pseudogenes is 5 times higher than ENSEMBL that used 2 FPKM as using small reads with a mismatch error rate can cause misalignment. It would be useful to perform a range of cutoffs from 0.3 FPKM, 0.6 FPKM and 1 FPKM and 2 FPKM and look at the distribution of detected pseudogenes .

*Response: Thank you very much for your helpful comments. To determine the distribution and sensitivity of detected pseudogenes under different cutoffs, we set a range of gradually increased FPKM cutoffs from 0.1 to 2. As shown in the updated Figure 2g, 26.3% of human and 28.2% of mouse pseudogenes were detected using FPKM ≥ 1, and 16.8% of human and 17% of mouse pseudogenes were detected using FPKM ≥ 2, higher than ENSEMBL (10% in human and 5% in mouse.*

Comment: It would be interesting if the isoseq confirmed pseudogenes can be identified as transcribed using the higher 1 or 2 FPKM threshold and using only the short read transcriptomic data as a control .

*Response: As you have expected, the pseudogenes detected by Iso-seq (median FPKM value, 8.5) showed significantly higher expression level than other pseudogenes (median, 0.33) (Wilcoxon test, P = 2.2×10-16). Using FPKM ≥ 1 as cutoff, 80% (140) of Iso-seq detected pseudogenes were identified as transcribed, which still remarkably higher than other pseudogenes (28% of them were transcribed) (Fisher exact test, P = 2.2×10-16). We added the results in manuscript (page 8, lines 171-173 and page 9, lines 182-184) and added the Figure 2f to illustrate the higher expression level of Iso-seq detected pseudogenes than that of others.*

Comment: The publication cited for the use of FPKM 0.3 actually in the method it states "the pseudogenes with detectable expression were defined as those with an average RPKM ≥ 1 across all samples in each cancer type, as used in the literature" so is more stringent cutoff than has been used for this analysis

*Response: Thanks for your careful check. Strangely, we found the publication (Han et al) we cited for the use of FPKM 0.3 has two versions in the method section. One says RPKM ≥ 1 (https://www.ncbi.nlm.nih.gov/pmc/articles/PMC4339277) as you found (Figure R2), while the other says RPKM ≥ 0.3 (from https://www.nature.com/articles/ncomms4963#Sec8) (Figure R3).*

Comment: and I would recommend the authors use a more stringent cutoff

*Response: We agree that a more stringent cutoff would be better and has applied FPKM ≥ 1 as cutoff for expression analysis in the updated version.*

Comment: 5. It would be useful to reproduce figure S8 with different RPKM cutoffs to see whether the distribution of expressed pseudogenes changes across biotypes when a more stringent FPKM is used as there are only 243 unitary pseudogene and yet it appears that nearly over 120 of these are transcribed and can be detected yet only 1 unitary pseudogene was detected using isoseq

*Response: Thanks very much for your suggestions. As you suggested, we have reproduced the figure with a wide range of FPKM cutoffs (now in Figure S9). The proportion of expressed unitary pseudogenes was always higher than that of other biotypes of pseudogenes under different cutoffs.*

*We also displayed the number of mouse pseudogenes detected by Iso-seq subdivided by biotypes (Figure 2c). A total of 7 mouse unitary pseudogenes were detected by Isoseq, accounting for 9% of all unitary pseudogenes (74 unitary pseudogenes were annotated in mouse genome), and the proportion was still higher than that of other biotypes of pseudogenes (4.5%, 1.1%, and 1.6% for polymorphic, processed, and unprocessed pseudogenes, respectively) (also see in Table S7). Among these 7 pseudogenes, 6 of them were annotated as “transcribed_unitary_pseudogenes” (1700086L19Rik, 1700031M16Rik, 4930451G09Rik, Thap6, 4932431P20Rik, Gm4275) by GENCODE and the last one was “unitary_pseudogene” (Gm49299). Consistent with GENCODE annotation, these 6 “transcribed_unitary_pseudogenes” were also expressed in our dataset, with maximum FPKM value 168.517, 388.163, 69.027, 6.850, 21.393, and 73.909, respectively, exhibiting higher expression levels than the last “unitary_pseudogene” (Gm49299) (maximum FPKM, 1.5).*

Comment: 6. The comment on Pg 10 line 213 " the highest pseudogene expression was in testis" maybe should be clarified as do the authors not attribute this to leaky expression and potentially not biologically relevant since "the massive chromatin remodeling during spermatogenesis causes leaky expression" (Soumillon et al. 2013 especially since a low FPKM was used for detection.

*Response: Thanks very much for your comments. We have added this article to our reference and added the sentence “which might be due to the leaky expression caused by extensive chromatin remodeling [39]” on page 11 lines 231-232 to clarify this possibility.*

Comment: 7. The graph S17 that indicates that dynamic developing pseudogenes are longer than non DDPs looks not very convincing S18-19 as the majority overlap in size,

*Response: Although the length distribution of DDPs and non DDPs were largely overlapped, there was statistically significant differences between them. For example, the median length of DDPs in human (shown in figure 5a) was 800 bp, while median length of non DDPs was only 513 bp (Wilcoxon test, p-value < 2.2×10-16). Also, in mouse, the median length of DDPs and non DDPs was 698 and 538 bp, respectively (Wilcoxon test, p-value < 2.2×10-16) (figure S17).*

Comment: Were any of the isoseq sequences identified as DDPs

*Response: Yes, among the 177 Iso-seq detected pseudogenes, there was a significant enrichment of DDPs (134, 76%) ( Fisher’s exact test, p-value < 2.2×10-16).*

Comment: and can the authors show the actual increase in TFs /extension or preserved polyA *sites/signals?*

*Response: Sure, we have exhibited an actual increase of TF binding sites at the promoter region of a DDP, 1700031M16Rik, and also displayed the consolidated poly A signal at the same loci (figure S21).*

Comment: It would also be useful to see which biotype category the DDPs are subcategorized into, eg are they mostly processed pseudogenes?

*Response: Yes, DDPs are mostly processed pseudogenes. We have found that 2,334 (60.5%) human DDPs and 2,507 (77.5%) mouse DDPs are processed pseudogenes. We have added this result to manuscript on page 13 (lines 269-270) as below.*

*“The majority of the DDPs were processed pseudogenes, accounting for 60.5% (2,334) human DDPs and 77.5% (2,507) mouse DDPs.”*

*Following your comment 9, we have also summarized this result in Supplementary Table S7).*

Comment: 8. Brosch et al identified 9 processed pseudogenes mouse using mass spec data from peptide atlas that is publicly available, so why could the authors not use this data to screen their pseudogenes ? Also in the 19% pseudogenes expressed do the 9 processed pseudogenes from Brosch et al appear ?

*Response: We thank the reviewer for the interesting idea. We scrutinized this paper (Brosch et al) and their supplementary materials, only 1 of 9 processed pseudogenes was shown with its name: OTTMUSG00000008151, ENSEMBL ID: ENSMUSG00000083780, and gene name: Gm12728 (from the link: https://vega.archive.ensembl.org/Mus_musculus/Gene/Summary?db=core;g=OTTMUSG00000008151;r=4:105789869-105794711). This pseudogene could generate 3 transcripts (ref: http://mar2017.archive.ensembl.org/Mus_musculus/Gene/Summary?g=ENSMUSG00000083780;r=4:105789869-105794711), the first two were annotated as mRNA based on HAVANA manual annotation, and the third one was translated processed pseudogene (OTTMUST00000018507.1), which Brosch et al found could encode peptide. We applied the coding potential calculator (CPC2 and CPAT) with default parameters and this transcript was predicted as noncoding RNA. We have also sent a query email to the corresponding author while have not received the response.*

*We thus have searched for other publicly available mass spec resources. As you have mentioned above, the annotation of pseudogenes and their biotypes was performed by GENCODE. They annotated 4 human translated pseudogenes (AC113404.3, PMS2P2, AC092128.1, CYP2G1P) using mass spectrometry data. A recent study (Wang et al, PMID: 30777892) generated a quantitative proteome across 29 human tissues and detected peptide evidence of 4 pseudogenes (WASH9P, GPX1, GBA3, and PNLIPRP2). Among the 8 translated pseudogenes with peptide evidence, 5 (63%) of them were determined as translated in our analysis (AC113404.3, CYP2G1P, GPX1, GBA3, and PNLIPRP2). We have added these results to our manuscript on page 18 (lines 375-389). Due to the current limited coverage and resolution, mass spectrometry (MS) might detect a limited subset of translated pseudogenes. We have pointed out the shortage and future direction in the discussion section (page 21, lines 447-450) as below.*

*“With the increased resolution, sensitivity, and sample size (including more tissues and developmental stages) in the future, MS technique could be used to systematically determine the translation ability and products of pseudogenes.”*

Comment: 9. What would help the readers navigate all the complex work that the authors have done would be a summary table of the number of mouse and mouse pseudogenes analysed by the different analyses (RNAseq, isoseq, Riboseq, CPAT) and actual number of pseudogenes detected (subdivided by biotypes) as it would be interesting to identify if the majority of DDPs were unprocessed pseudogenes and what percentage of processed pseudogenes were translated etc.

*Response: Thanks very much for your advice. We have summarized the number of pseudogenes (GENCODE annotation), expressed pseudogenes (FPKM ≥ 1), Iso-seq detected pseudogenes, DDPs, predicted pseudogenes (CPAT & CPC2), and Ribo-seq validated pseudogenes (subdivided by biotypes and species) in Supplementary Table S7.*

Comment: Minor points :

1. Pg4 line 72 processed Adh gene (jingwei), is there a reference missing here ?

*Response: At the end of this sentence, we cited two papers which determined processed Adh gene (jingwei) as a functional copy. To make it clearer, we have moved the two references to the end of "processed Adh gene (jingwei)" on page 4 (lines 73-74) as below.*

*“Pioneering work has rehabilitated a processed Adh gene (jingwei) as a functional copy [10, 11], instead of a defective pseudogene as previously reported.”*

Comment: 2. Pg4 line 78 please expand what is refereed to concerning ceRNA behaviour.

*Response: We have expanded what is refereed to concerning ceRNA behaviour (pages 4-5, lines 85-88) as below.*

*“Also, most of them were limited to the interplay between pseudogenes and their parent coding genes like competing endogenous RNAs (ceRNA) behavior, where pseudogene transcripts could regulate their parent mRNAs by competing for identical microRNAs [18, 24].”*

Comment: 3. page 7 line 142 why the publication by Troskie et al 2021 support issue of short reads hindering identifying transcribed pseudogenes as is about long reads

*Response: We have changed the reference to Cheetham et al 2020, which reviewed the strength and shortage of different high-throughput methods for detecting pseudogenes.*

Comment: 4. Pg 20 line 433 typo Pipeline

*Response: We have changed “Pipline” to "Pipeline". Thank you again for all the constructive comments and suggestions.*

**Second round of review**

**Reviewer 1**

The authors have greatly improved their manuscript and addressed many of my concerns. The article contributes to the growing body of evidence of mammalian pseudogene function and the analysis of dynamic pseudogene expression during development is novel. However, prior to publication it is essential that the authors exclude mismapping artifacts from their datasets.

Major comments:

1. FigS3 depicts the correlation of expression between pseudogenes and their parent genes. This essential to understand how accurate the mappings are likely to be. However, this analysis should also be conducted on just expressed pseudogenes or DPPs. As most pseudogenes will have no expression the current analysis is biased towards showing a lack of correlation. DPPs with a high correlation with their parent gene should be excluded from further analyses as these are likely to be attributable to mis-mapping. A cursory look at the pseudogenes that were detected by Iso-Seq showed that many of these pseudogenes have a very high percentage identity with their parent genes (> 99% for several examples I looked at). These pseudogenes are very prone erroneous mappings particularly with the short reads used in subsequent analyses. The authors should take great care, including manually examining alignments at representative loci to ensure that there is not extensive misalignment.

2. I could not find a table of the expression of DPPs in the various datasets. This information is essential for the paper to be of broad use to researchers.

Minor comments:

The text states that FER1L4 is a DPP. Current GENCODE annotations list this as a protein-coding gene. A different example should be used to illustrate this point.

4632415L05Rik is 99.8% identical to the parent gene.

**Reviewer 2**

I would like to thank the authors for their thorough responses to my review questions and agin this is a large amount of experimental and bioinformatics analysis that has been done examining expressed pseudogenes . They authors appeared to have addressed the majority of my concerns and have updated the manuscript, where appropriate and also updated the figures. The updated supplementary tables are useful to navigate the overview of the data alongside the GENCODE biotypes and I have no further comments concerning the manuscript.

**Authors Response**

**Point-by-point responses to the reviewers’ comments:**

Reviewer #1:

Comment: The authors have greatly improved their manuscript and addressed many of my concerns. The article contributes to the growing body of evidence of mammalian pseudogene function and the analysis of dynamic pseudogene expression during development is novel.

*Response: We sincerely appreciate your positive remarks on the manuscript.*

Comment: However, prior to publication it is essential that the authors exclude mismapping artifacts from their datasets.

*Response: Thank you very much for your constructive comments, which we have addressed in details as below.*

Major comments:

Comment: 1. FigS3 depicts the correlation of expression between pseudogenes and their parent genes. This essential to understand how accurate the mappings are likely to be.

*Response: We agree with the reviewer that mapping uncertainty could be an issue, and have taken care of our mapping method. One key reason that we applied the software STAR to read alignment is its accuracy in distinguishing similar paralogs. Ma et al (PMID: 33476555) simulated RNA-seq reads derived from these coding paralogs and confirmed the great performance of STAR in pseudogene alignment when the identity is lower than 96%. We further analyzed the identity and found that more than 97.7% of all pseudogenes have the identity low than 96% with their coding paralogs (Figure S33), supporting the rationality of using STAR in our alignment.*

*We have also added the justification for our methods (page 25, lines 532-533) as below.*

*“we applied STAR (v2.6.1) [75], which is accurate in distinguishing similar paralogs [21] (Additional file 1: Figure S33-S35), ”*

Comment: However, this analysis should also be conducted on just expressed pseudogenes or DPPs. As most pseudogenes will have no expression the current analysis is biased towards showing a lack of correlation.

*Response: Thanks very much for your suggestions. We have conducted the same analysis on both expressed pseudogenes and DDPs. For expressed pseudogenes and their parent coding genes, the median observed positive and negative correlation coefficient (R) is 0.31 and -0.08, respectively. Meanwhile, for DDPs and their parent genes, the median observed positive and negative correlation coefficient (R) is 0.33 and -0.08, respectively. We have added the results in the manuscript (page 9, lines 176-180 and page 13, lines 277-281) as below.*

*“We also analyzed the expression correlation between DDPs and their parent coding genes (Additional file 1: Figure S18) and found that the median positive and negative correlation coefficient (R) was 0.33 and -0.08, respectively.”*

Comment: DPPs with a high correlation with their parent gene should be excluded from further analyses as these are likely to be attributable to mis-mapping.

*Response: Thank you. Following your advice, we have excluded 254 DDPs that showed a high correlation (R ≥ 0.6) with their parent coding cognates from further analyses (page 13, lines 280-281). We have updated all results (Figure 2-7) and found consistent patterns with those before the exclusion (e.g. high conservation, specific and dynamic expression, active regulation, and involvement in cancer transcriptome changes).*

Comment: A cursory look at the pseudogenes that were detected by Iso-Seq showed that many of these pseudogenes have a very high percentage identity with their parent genes (> 99% for several examples I looked at). These pseudogenes are very prone erroneous mappings particularly with the short reads used in subsequent analyses. The authors should take great care, including manually examining alignments at representative loci to ensure that there is not extensive misalignment.

*Response: As you suggested, we have also manually examined the alignments at representative loci and found that the reads were precisely aligned. We also used the RNA-seq reads derived from pseudogene loci to run BLAST and found that the average identity of reads at pseudogene loci was 99.7%, while the average identity at other regions was 84.6% (Figure S34), suggesting that there was no extensive misalignment.*

Comment: 2. I could not find a table of the expression of DPPs in the various datasets. This information is essential for the paper to be of broad use to researchers.

*Response: Thank you very much for your advice. We have added the expression level of human and mouse DDPs in developmental transcriptome, GTEx project, ENCODE project, and a dataset of 32 human adult tissues to Supplementary Table S10-14.*

Minor comments:

Comment: The text states that FER1L4 is a DPP. Current GENCODE annotations list this as a protein-coding gene. A different example should be used to illustrate this point.

*Response: We thank the reviewer for pointing this out. We showed the expression level of another DDP, ABCC13, in colorectal cancer and normal adjacent tissue (page 20, lines 432-435, Figure S32) as below.*

*“An example of upregulated DDPs was ABCC13, which exhibited remarkably a higher expression level in colorectal cancer than in normal adjacent tissue (Additional file 1: Figure S32).”*

Comment: 4632415L05Rik is 99.8% identical to the parent gene

*Response: Thank you. 4632415L05Rik is an unprocessed pseudogene, which could generate two transcripts. The longer one (ENSMUST00000198527.4) is 6,147 bp, spanning the entire gene body region, and the shorter one (ENSMUST00000059704.5) is 1,027 bp. We used the two transcripts to run BLAST against the genome sequence excluding the 4632415L05Rik loci and found that the identities were 91% (matched sequence, 847 bp, 14% of the entire transcript) and 85.6% (matched sequence, 520 bp, 51% of the entire transcript), less than above cutoff (96%). We also checked the RNA-seq reads derived from 4632415L05Rik and found that the average number of mismatch at 4632415L05Rik was 0.08 while the average number of mismatch at other regions (second-best hit) was 8.04 (Figure S35), suggesting that our alignment was precise.*

*We thank the reviewer again for his/her efforts and constructive suggestions.*

Reviewer #2:

Comment: I would like to thank the authors for their thorough responses to my review questions. They appeared to have addressed the majority of my concerns and have updated the manuscript where appropriate and also updated the figures. The updated supplementary tables are useful to navigate the overview of the data alongside the GENCODE biotypes and I have no further comments concerning the manuscript.

*Response: Thanks again for all the constructive comments and suggestions.*

**Third round of review**

**Reviewer 1**

In their revised manuscript the authors have addressed my concerns regarding read-mismapping. As such, I now support publication of the manuscript. The authors are to be congratulated on their novel contribution to pseudogene functionality.
